# New Chlormequat-Based Ionic Liquids as Plant Resistance Inducers

**DOI:** 10.3390/molecules30214203

**Published:** 2025-10-27

**Authors:** Rafal Kukawka, Maciej Spychalski, Patrycja Czerwoniec, Beata Hasiów-Jaroszewska, Sylwia Stępniewska-Jarosz, Emilia Frydrych-Tomczak, Marcin Smiglak

**Affiliations:** 1 Poznan Science and Technology Park, Adam Mickiewicz University Foundation, Ul. Rubiez 46, 61-612 Poznań, Poland; 2 ATI Sp. z o.o., Ul. Rubież 46, 61-612 Poznań, Poland; 3 Faculty of Chemistry, Adam Mickiewicz University, Ul. Uniwersytetu Poznańskiego 8, 61-614 Poznań, Poland; 4 Institute of Plant Protection—National Research Institute, Ul. Wł. Węgorka 20, 60-318 Poznań, Poland

**Keywords:** plant protection, systemic acquired resistance, ionic liquids, bifunctionality, biological activity, plant resistance inducers, 2-chloroethyltrimethylammonium cation, chlormequat, *Tobamovirus tabaci*

## Abstract

Active compounds used in agriculture are mainly in the form of acids. This applies primarily to substances that are inducers of systemic acquired resistance, which is one of the most promising methods of supporting plants in the fight against pathogens. The physicochemical properties and biological activity of such substances can be improved by derivatizing them to salt forms. We used the concept of ionic liquids to obtain novel compounds in the form of chlormequat ionic liquids. In this study we present synthesis and characterization of a series of novel ionic liquids composed of the chlormequat cation paired with plant resistance-inducing anions, including salicylic acid and its chlorinated derivatives, nicotinic acid and isonicotinic acid. The results indicate that the new compounds in the form of salts are characterized by better biological activity related to SAR induction and lower phytotoxicity compared to the parent compounds as their equivalents in acid forms. The obtained compounds demonstrated the ability to activate defense responses in tobacco and to reduce susceptibility to viral infection, highlighting their potential for further application in crop protection.

## 1. Introduction

One of the core principles of Green Chemistry is to design safer chemicals—that is, to develop substances with minimal toxicity while preserving their intended biological activity. This principle is particularly relevant in agriculture that uses herbicides, fungicides, insecticides, and plant growth regulators through which chemicals are introduced into complex biological systems [1,2,3]. These agents affect both target and non-target organisms, and thus designing next-generation agrochemicals requires a careful balance between biological efficacy and environmental and human safety [4].

A promising strategy in plant protection is the use of systemic acquired resistance (SAR) inducers, which align with sustainable chemistry principles. Instead of targeting pathogens directly, SAR inducers activate the plant’s innate immune system by increasing the production of endogenous signaling molecules such as salicylic acid and upregulating pathogenesis-related (PR) proteins [5,6]. Since plant defense mechanisms are typically initiated only after pathogen recognition, which is often too late to prevent damage, SAR inducers can prime plants’ immune systems in advance, enabling a more efficient response upon infection [7]. The group of known SAR inducers include salicylic acid and its analogs [8,9,10,11,12]; benzothiadiazoles and their analogs [13,14,15]; and heteroaromatic carboxylic acids like nicotinic and isonicotinic acid [16]. In general, SAR inducers are effective at much lower application rates than traditional pesticides. For instance, similar effects in terms of SAR induction on sugar beet plants were observed after the application of the SAR inducer named 3,5-dichlorosalicylate cholinum, which was applied in a total dose of 320 g/ha after the application of active substances present in fungicides for a total dose of 600 g/ha.

Moreover, SAR inducers typically exhibit low toxicity toward non-target organisms, including aquatic organisms [17], pollinators and beneficial soil microbiota [18], and often display favorable degradation and residue profiles [17], reducing environmental persistence and meeting both regulatory standards and consumer expectations for safer crop protection. However, their wider application is constrained by poor aqueous solubility, which impairs formulation stability, foliar absorption, and systemic translocation. Formulations often require additional additives such as thickeners or dispersants, increasing the number of chemical components and making the formulation process more complex [19]. Additionally, their poor solubility restricts systemic mobility within plants, diminishing effectiveness in field conditions [20].

To improve solubility, such inducers are commonly converted into sodium salts—a standard agricultural practice (e.g., sodium benzoate, sodium 2,4 D, sodium glyphosate) [21,22]. While sodium salts enhance aqueous solubility, they are highly hydrophilic and poorly lipophilic, which may limit uptake through plant cuticles. Additionally, excess sodium ions (Na^+^) can disrupt nutrient homeostasis—particularly through antagonizing potassium (K^+^)—and interfere with calcium (Ca^2+^) and magnesium (Mg^2+^) absorption, leading to adverse physiological effects [23,24]. Likewise, chloride ions (Cl^−^), typical of salts such as chlormequat chloride, may hinder nitrate uptake, disrupt ionic balance, induce chlorosis or necrosis, and acidify the rhizosphere—negatively impacting microbial communities and plant health [25].

Chlormequat chloride ([CC][Cl]) is a quaternary ammonium salt of a structure similar to that of choline chloride that is a metabolite important for membrane stabilization, osmoregulation, and synthesis of stress-protective compounds like glycine betaine [26]. Previous studies on choline-based ionic liquids have shown that their application has brought about enhanced germination, increased levels of growth-promoting hormones, and low phytotoxicity in maize seedlings [27].

Over the past few decades, ionic liquids (ILs) have emerged as a rapidly growing area of research. These substances are organic salts with melting points typically below 100 °C, which gives them a set of unique physicochemical features [28]. A key advantage of ILs lies in the possibility of independent selection of cations and anions, allowing the rational design of compounds with properties suited to specific tasks. In 2007, the Rogers group introduced the concept of “third-generation ionic liquids,” emphasizing the intentional construction of salts with dual functions, first illustrated using pharmaceutically active compounds [29]. Since then, this concept has been successfully extended to other disciplines, including agriculture and crop protection [30]. Their ionic nature provides several benefits for use as plant protection products (PPPs). On the one hand, low volatility together with high thermal and chemical stabilities make their application in greenhouses and field conditions particularly safe. On the other hand, the modularity of ILs enables fine-tuning of their performance: a given compound can retain the same primary ion yet exhibit different physical behavior simply through modification of the counterion [31,32].

Given its agricultural relevance and ionic structure, the chlormequat cation (2-chloroethyltrimethylammonium cation, [CC]^+^) presents an attractive scaffold for designing multifunctional ionic liquids (ILs) incorporating resistance-inducing anions. Recently, ILs have emerged as promising agrochemical formulations, offering tunable physicochemical properties, reduced volatility, and potential multifunctionality [1,2,33]. While ILs have been successfully explored for herbicides and plant growth regulators, their application as SAR inducers remains largely unstudied.

Studies exploring SAR-inducing compounds paired with choline or related quaternary ammonium cations revealed their enhanced biological activity compared to parent acid forms [34], suggesting that the chlormequat cation ([CC]^+^) could be an effective scaffold for bifunctional agrochemicals. Given its ionic structure and agricultural relevance, [CC]^+^ is a suitable candidate for constructing ionic liquids (ILs) with resistance-inducing carboxylate anions. Such ILs have the potential to overcome solubility and formulation problems associated with conventional salts—while reducing the accumulation of problematic Na^+^ or Cl^−^ ions in agroecosystems.

In this study, we synthesized and characterized a series of novel ionic liquids composed of the chlormequat cation paired with plant resistance-inducing anions, including salicylic acid and its chlorinated derivatives, nicotinic acid and isonicotinic acid.

We hypothesize that the investigated chlormequat salts are capable of enhancing systemic acquired resistance (SAR) induction (manifested as reduced susceptibility to viral infection) while simultaneously reducing the risk of phytotoxic effects compared to those of the parent compounds in the form of acid. The experimental setup of compounds subjected to study allows analysis of both their physicochemical and biological properties in chlorinated and non-chlorinated analogs in both acid and ionic liquid forms. This facilitates the development of safer and more effective agrochemical agents in line with sustainable crop protection approaches.

## 2. Results

**Figure 1:**
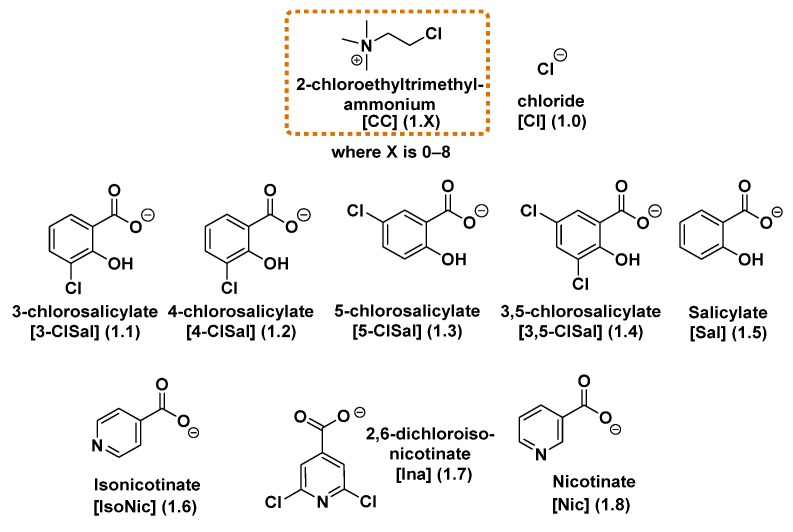
Structures of chlormequat-based ILs.

We successfully synthesized eight salts based on the chlormequat cation combined with plant resistance-inducing anions derived from salicylic, nicotinic, and isonicotinic acids. All selected acids have been previously reported in the literature as potential systemic acquired resistance (SAR) inducers, with chlorosalicylate anions in particular known for their ability to trigger plant defense responses [8]. Two of the synthesized salts—chlormequat salicylate and nicotinate [35]—have been previously reported by other research groups but have not been investigated as plant resistance inducers. The remaining six compounds are new chemical compounds, not described in the literature to date. The chemical structures of all synthesized salts are shown in Figure 1.

### 2.1. Thermal Properties and Glass Transitions

Differential scanning calorimetry (DSC) was used to determine the melting points and glass transition temperatures (T_g_) of the synthesized chlormequat-based salts (Table 1). Four compounds, [CC][Sal], [CC][IsoNic], [CC][Ina], and [CC][Nic], met the thermal definition of ionic liquids, exhibiting melting points below 100 °C: 70.3 °C (1.7), 85.3 °C (1.6), 89.9 °C (1.8), and 93.3 °C (1.5). The remaining salts, [CC][3-ClSal], [CC][4-ClSal], [CC][5-ClSal], and [CC][3,5-ClSal], had melting points above 100 °C, indicating their more crystalline character.

All synthesized salts displayed substantially lower melting points than the [CC][Cl] precursor (240 °C, with concurrent decomposition), consistent with reduced lattice energy after substituting chloride with bulkier, organic anions. Glass transition phases were observed in all ionic liquids and most salts, with T_g_ values ranging from 1.3 °C ([CC][3-ClSal]) to 35.6 °C ([CC][IsoNic]). The salts containing salicylate anions generally had lower T_g_ values compared to those with nicotinate or isonicotinate anions. Notably, compound [CC][Ina] had the lowest melting point and a relatively high T_g_ (30.6 °C), indicating unique structural packing effects.

Thermogravimetric analysis (TGA) revealed that the synthesized salts exhibited decomposition onset temperatures (T_onset_) ranging from 199.8 °C to 234.6 °C, with 5% weight loss onset temperatures (T_5%onset_) between 166.9 °C (**1.8**) and 217.1 °C (**1.3**). Thermal stability was strongly influenced by the nature of the anion. The salts containing nicotinate ([CC][Nic]) or isonicotinate ([CC][IsoNic] and [CC][Ina]) anions showed the lowest stability, whereas the chlorinated salicylate derivatives—particularly [CC][5-ClSal] (**1.3**)—were the most thermally robust.

Compared to [CC][Cl] (T_onset_ = 259.8 °C), all synthesized salts had reduced thermal stability, a common effect of replacing inorganic anions with organic ones. TGA thermograms confirmed a single-step decomposition process for all compounds, suggesting that they are suitable for formulation processes conducted at moderate temperatures, but may require stabilization for applications involving prolonged exposure to heat.

### 2.2. Solubility

The solubility of the synthesized ILs was evaluated in six representative solvents covering a polarity range from high to low. The obtained data are summarized in Table 2.

All products readily dissolved in highly polar solvents, including water and methanol. None of the studied derivatives dissolved in acetone, including chlormequat chloride and its salicylate, chlorosalicylate and nicotinate salts. This lack of solubility can be attributed to the insufficient polarity of acetone to provide effective solvation of the highly ionic structures, which results in unfavorable interactions between the solvent molecules and the charged species. In contrast, solubility differences were observed in isopropanol. Chlormequat chloride remained insoluble, whereas its ionic liquid salts with salicylic and chlorosalicylic acids dissolved. The results observed in solvents of the lowest polarity support the general assumption that ionic liquids are poorly miscible with liquids exhibiting low dielectric constants [36]. Accordingly, none of the ILs dissolved in hexane (εr = 1.9) or in toluene (εr = 2.4).

### 2.3. Phytotoxicity

Preliminary assessment of the phytotoxicity of the tested compounds was performed on radish sprouts. Phytotoxic effects manifest as a reduction in the mass of sprouts compared to that of untreated control plants. The average masses of 25 radish sprouts resulting from the application of tested variants of treatment are indicated in Table 3.

The first approach to the analysis of the obtained results involves verification of the hypothesis concerning the reduction in the phytotoxic effect on sprouts caused by a compound in the form of a salt in relation to that caused by the equivalent of this compound in acid form.

The results obtained for the group of compounds containing 3-chlorosalicylic acid and its salt derivative indicate that in the case of both tested concentrations, significantly higher sprout mass was achieved for the treatment with the substance in the form of a salt. Results obtained for the group of compounds containing 4-chlorosalicylic acid, 5-chlorosalicylic acid, 3,5-dichlorosalicylic acid, salicylic acid, 2,6-dichloroisonicotinic acid and their salt derivatives are in line with results obtained for the 3-chlorosalicylic acid and its salt. As for the group of compounds containing isonicotinic acid and its salt derivative, the results follow a similar trend, although the difference between the results obtained for the treatment with both these compounds at the concentration of 125 mg/L is not statistically significant. As for the group of compounds containing nicotinic acid and its salt derivative, no significant differences were observed when comparing the effects of both these compounds when applied at concentrations of either 125 mg/L or 250 mg/L.

Another approach to analyzing the obtained results involves a comparison of the phytotoxic effect of the tested substances performed separately for each of the concentrations used. To facilitate this analysis, the results were converted from the mass of 25 sprouts treated according to each variant of treatment to the percentage of reduction in sprout mass in comparison to that of untreated control plants (Table 4).

The results obtained for both concentrations of active substances used in this study follow a similar trend. In general, treatment with substances in the form of acid resulted in a higher reduction in sprout mass compared to that resulting from the treatment with substances in the form of salt. The substance causing the greatest phytotoxic effect, manifested as the highest mass reduction compared to that of the untreated control, was 3-chlorosalicylic acid. However, this substance and its salt derivative were among the substances with the highest efficiency related to SAR induction. On the other hand, nicotinic acid and its equivalent in the salt form are among the substances causing the lowest sprout mass reduction, but their SAR induction efficiency is also moderate.

### 2.4. SAR Induction Activity

All eight salts demonstrated SAR induction properties, as shown by a reduction in the necrotic area caused by viral infection. The percentage of necrotic area on leaves of plants treated with the tested active compounds is shown in Table 5 and in Table S1.

The first approach to analysis of the obtained results involves verification of the hypothesis concerning the reduction in the degree of infection of plants treated with a given compound in the form of a salt in relation to the leaf infection obtained after treating the plants with the equivalent of this compound in the acid form. The results obtained for the group of compounds containing 3-chlorosalicylic acid and its salt derivative indicate that in the case of both tested concentrations specified in the table, lower leaf infection was achieved for the plants treated with the substance in the form of salt, although the differences were not confirmed statistically. As for the group of compounds containing 4-chlorosalicylic acid and its salt derivative, it was observed that application of the salt derivative resulted in lower leaf infection compared to that resulting from the application of the acidic form; however, the difference was significant only when the tested substances were applied at a concentration of 125 mg/L. The results obtained for the plants treated with 5-chlorosalicylic acid and [CC][5-ClSal] followed the same pattern as those obtained for the group of compounds containing 3-chlorosalicylic acid and its salt derivative. As for the treatment with 3,5-dichlorosalicylic acid and its salt derivative, significantly lower leaf infection was observed after the application of the salt derivative only at the concentration of 125 mg/L. Regarding the results obtained for the group of compounds containing salicylic acid and its salt form, significantly lower leaf infection was obtained as a result of application of [CC][Sal] at both concentrations tested. As for the treatment with 2,6-dichloroisonicotinic acid and its salt derivative, lower leaf infection was observed for the plants treated with the salt derivative; however, the difference was not statistically proven. As for the group of compounds containing isonicotinic acid and its salt derivative, lower leaf infection was observed for [CC][Isonic] only at the concentration of 125 mg/L. The results obtained for the group of compounds with nicotinic acid and its salt form indicated a significantly lower leaf infection after treatment with [CC][Nic] at both concentrations tested.

Another approach to analysis of the obtained results involves a comparison of the efficiency of the tested substances performed separately for each of the concentrations used. To facilitate this analysis, the results were converted from the percentage of necrotic area covering leaves of plants treated according to each variant of treatment to the percentage reduction in necrotic area in comparison to that of untreated control plants (Table 6).

**Figure 2:**
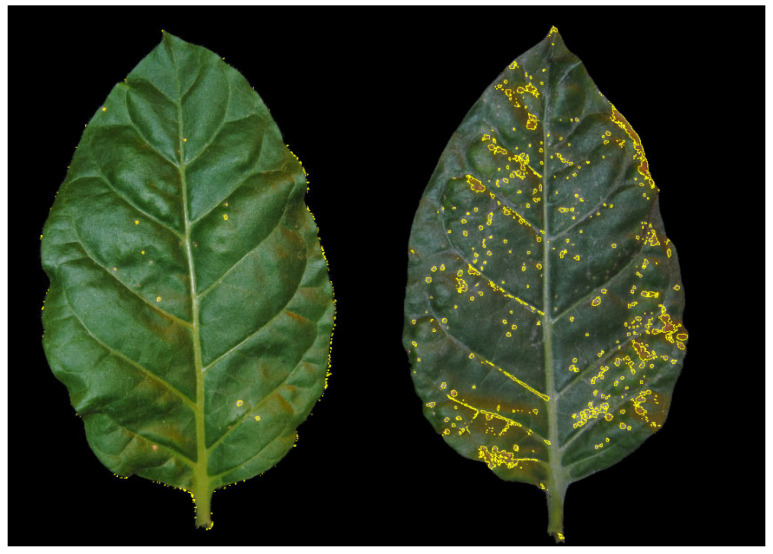
Reduction in the number of viral necrotic spots resulting from the application of the SAR-inducing salt [CC][3-ClSal] at a concentration of 250 mg/L (**left**), compared with the control (water (**right**)). Viral necrotic spots on tobacco leaves were assessed seven days after inoculation. Mechanical inoculation was performed 7 days after spraying. Necrotic spots (outlined in yellow) were marked using ImageJ software 1.53m.

The results obtained for both concentrations of active substances used in this study follow a similar trend. For both tested concentrations, the substances with the best SAR induction properties were [CC][3-ClSal] (Figure 2), [CC][5-ClSal] and their equivalents in the form of acid, i.e., 5-chlorosalicylic acid and 3-chlorosalicylic acid (Figures S1–S9). In turn, the substances with the worst SAR induction properties were salicylic acid, nicotinic acid and isonicotinic acid, i.e., parent compounds not subjected to any derivatization.

As to the differences between the effects of applications of the substances studied at the two concentrations, the general trend indicates that the greater reduction in necrotic area was observed for the concentration of 250 mg/L compared to that obtained after the use of a concentration of 125 mg/L. However, this difference was not statistically significant for every substance (Table 4). The difference in the percentage reduction in leaf necrotic area observed for the compounds at the two concentrations used in this study was smaller for the substances with higher SAR induction efficiency. For example, the difference between [CC][5-ClSal] applied at a concentration of 125 mg/L and that at 250 mg/L was approximately 4.5 percentage points. However, for substances with weaker SAR induction efficiency, the differences between the compounds’ effects at two concentrations were greater. For example, for [CC][Nic] the difference in its effects at concentrations of 125 mg/L and 250 mg/L was approximately 22 percentage points.

## 3. Discussion

In this study, we identified ionic liquids derived from the chlormequat cation paired with chlorinated and dichlorinated salicylates as promising candidates for the development of novel plant resistance inducers. These compounds demonstrated the ability to activate defense responses in tobacco and to reduce susceptibility to viral infection, highlighting their potential for further application in crop protection. Although the use of ionic liquids as plant resistance inducers has not been widely described in the literature, valuable insights can be gained by comparing the physicochemical properties and biological activity of these compounds with neutral salicylic acid derivatives, which have been studied as model structures for chemical modification. Such comparisons provide a basis for understanding how ionic transformation may further modulate biological activity.

### 3.1. Properties of the Obtained Compounds

The obtained compounds were thermally stable salts with glass transition points between 1.3 °C and 32.2 °C for [CC][3-ClSal] and [CC][Nic], respectively. All products readily dissolved in highly polar solvents, including water and methanol, which is in agreement with earlier reports concerning plant resistance inducers or ionic liquid herbicides based on carboxylic acids [34,37,38] as an anion. Moreover, Amoore et al. [39] have demonstrated that the introduction of chlorine atoms into organic molecules frequently decreases their affinity towards water. This observation is consistent with our results, indicating that chlormequat-based ionic liquids form more soluble salts with salicylate than with monochlorinated salicylate or 3,5-dichlorosalicylate.

Chlormequat salicylate shows solubility above 100 g/L, whereas the salts containing chlorine-substituted anions display only moderate solubility, typically within the range of 33.3–100 g/L. Nevertheless, such levels are still sufficiently high to consider these compounds as suitable active ingredients in formulations designed to induce plant resistance. The presence of one or more chlorine atoms in place of hydrogen on the aromatic ring of the anion alters the solvation of ILs by water molecules, which in turn reduces their hydrophilic character.

### 3.2. Phytotoxicity

The experiment on radish sprouts was selected to preliminarily assess the phytotoxicity of the tested compounds. The effect of treatment with [CC][Cl] is treated as an indication of phytotoxicity of the anion used to obtain chlormequat ILs. The mass of radish sprouts was significantly lower compared to that of the control variant, for both concentrations of [CC][Cl]. After the treatment with [CC][Cl] at the concentration of 125 mg/L, the reduction in the radish sprout mass relative to the control variant was equal to 22%, while for the treatment at the concentration of 250 mg/L, it was equal to around 33%. Of note, the percentage reduction in radish sprout mass was among the lowest observed for chlormequat ILs. As for the group of compounds containing nicotinic acid and its salt derivative, chlormequat IL, no phytotoxic effect was observed for the treatment with [CC][Nic] at a concentration of 125 mg/L and nicotinic acid at a concentration of 125 mg/L or 250 mg/L, as the masses of radish sprouts treated according to indicated variants belong to the same statistical group. A significant decrease in the radish sprout mass was observed only when [CC][Nic] was applied at the concentration of 250 mg/L. It might be concluded that such a phytotoxic effect was due to the presence of the anion derived from [CC][Cl] rather than the cation from nicotinic acid.

In the case of 2,6-dichloroisonicotinic acid (INA), which was one of the first SAR inducers discovered but at the same time insufficiently tolerated by some crops, causing phytotoxic effects in plants [16], the corresponding substance in the form of chlormequat IL had a significantly lower toxicity than the equivalent of this substance in the form of salt. This confirms the hypothesis that derivatization to salt form results in lower phytotoxicity of the substance.

For instance, when considering a group of compounds containing 3-chlorosalicylic acid and its salt derivative, the mass of radish sprout treated with 3 chlorosalicylic acid at the concentration of 250 mg/L and [CC][3-ClSal] at the concentration of 125 mg/L can be assumed as being at a similar level, although significant differences between the indicated variants were observed. This is mainly due to the fact that the mass of the cation derived from the acid equivalent makes up approximately half of the molar mass of the compound in the form of chlormequat IL. The efficiency of SAR induction that resulted from the applications of both indicated treatment variants was at the same level. However, it should be noted that better SAR induction properties were observed for the higher of the concentrations tested, i.e., 250 mg/L, and generally for the variant with the compounds in salt forms, not the acid forms. At a concentration of 250 mg/L, a significantly weaker phytotoxic effect was observed for the compounds in the salt form than for those in the acid form.

Apart from the possibility of verifying our hypothesis, the phytotoxicity assays performed here also served as a criterion enabling the identification of candidate compounds with an acceptable balance between biological activity and plant tolerance. This step prevents the advancement of excessively toxic derivatives into more costly greenhouse or field trials.

### 3.3. SAR Induction

The *Tobamovirus tabaci* (tobacco mosaic virus, TMV)–*Nicotiana tabacum* cv. Xanthi model is widely used for studying SAR induction. This is due to the well-characterized host–pathogen interaction, the rapid and easily observable development of infection symptoms, and the high reproducibility of results [40,41]. Therefore, it serves as a standard tool for validating potential SAR inducers.

Virus diseases are significant threats to modern agriculture, and their control remains a challenge to the management of cultivation. Viral disease pandemics and epidemics were estimated to have a global economic impact of more than USD 30 billion annually [42]. As strict intracellular pathogens, they cannot be controlled chemically, and prophylactic measures consist mainly in the destruction of infected plants and excessive pesticide applications to limit the population of vector organisms. TMV belongs to the Tobamovirus genus and has been found to cause serious diseases in vegetables (especially in the Solanaceae family), tobacco, and many ornamental species. Tobamoviruses are different from most plant viruses because their virus particles remain infectious in relatively adverse environments outside the host plant for long periods. TMV is readily transmitted via various means such as soil, plant debris and seeds, and any minor damage to plants provides an entry point for TMV infection, further complicating the control efforts [43,44,45]. Therefore, there are great needs to develop methods to reduce the virus in the environment and induce plant immunity simultaneously.

The literature describes chemical modifications of salicylic acid at the carboxyl group (C1, e.g., esterification [46]), at the hydroxyl group (C2, e.g., acetylation or glucosylation [11,47]), or at positions C3–C6 of the aromatic ring through substitution with halogen, methyl, or hydroxyl groups [8,9,12,48]. Previous studies demonstrated that neutral derivatives such as acetylsalicylic acid (C2 modification) were effective against tobacco mosaic virus in tobacco [48]. Methyl salicylate (C1 esterification) has been identified as a crucial systemic signal in SAR, acting as a mobile form of salicylic acid that can be transported via the phloem and reconverted into the active compound in distal tissues [46]. Other modifications at C5, including 5-methylsalicylic acid, 5-fluorosalicylic acid, and 5-iodosalicylic acid, were effective in *Arabidopsis* against *Pseudomonas syringae* [9]. Silverman et al. (2005) systematically analyzed the structure–activity relationships of forty-seven mono- and multi-substituted salicylates, using PR-1 protein accumulation as a marker of SAR, which correlated well with resistance to TMV [48]. They demonstrated that halogenated analogs substituted in the 3- and/or 5-positions, particularly fluorinated and chlorinated derivatives, induced PR-1 expression more strongly than unsubstituted salicylic acid. Their reported potency decreased in the following order: 3-chlorosalicylate > 3,5-difluorosalicylate > 5-chlorosalicylate > 5-fluorosalicylate > 3,5-dichlorosalicylate > 4-fluorosalicylate > 3-fluorosalicylate > 3-chloro-5-fluorosalicylate > 4-chlorosalicylate > salicylic acid. This is consistent with our findings, as resistance-inducing activity was the highest for 3-chlorosalicylic acid, followed by 5-chlorosalicylic acid and 3,5-dichlorosalicylic acid, while 4-chlorosalicylic acid and salicylic acid showed substantially lower activity. The higher activity of mono- and di-halogenated salicylic acid derivatives compared with that of salicylic acid itself can be explained by their specific chemical interactions with proteins of the former. Protein–ligand interactions are determined primarily by electrostatic forces, hydrogen bonding, and van der Waals interactions, but halogen atoms can also form directional halogen bonds [9]. These bonds are comparable in strength and specificity to hydrogen bonds and can stabilize protein–ligand complexes [49]. Furthermore, halogen substitution may improve membrane permeability and prolong biological half-life by reducing metabolic degradation, thereby extending the time of its activity [9].

Neutral salicylic acid and its sodium salt both exhibit notable limitations when used as resistance inducers. Although sodium salicylate offers higher water solubility, its low lipophilicity restricts penetration through the cuticle, resulting in only modest activity in plants [9,50]. In contrast, ionic liquids containing the chlormequat cation combine aqueous solubility with moderate lipophilicity, thereby improving membrane permeability penetration and enhancing elicitor activity. In addition, the chlormequat cation is structurally related to choline, a metabolite involved in stress protection and osmoregulation, which may further contribute to the elicitor effect. In our study, the transformation of neutral acids into their ionic forms generally enhanced activity related to SAR induction. For instance, the reduction in necrotic spots, used as a measure of SAR activity, was higher in the plants treated with [CC][3-ClSal] and [CC][5-ClSal] compared to that in the plants treated with the corresponding neutral acids, although these differences were not statistically significant. A clearer effect was observed for [CC][3,5-diClSal], [CC][Sal], [CC][IsoNic], and [CC][Nic], whose conversion into the chlormequat ILs resulted in a statistically significant increase in SAR-inducing activity. The only exception was [CC][Ina], for which ionic transformation did not alter effectiveness.

All in all, conversion into the chlormequat ILs generally improved resistance induction relative to the corresponding neutral acids, confirming the advantage of the ionic liquid form. This interpretation is consistent with earlier observations that the more lipophilic acetylsalicylic acid induced greater PR-1 accumulation than the highly hydrophilic sodium salicylate, thus achieving higher effectiveness in SAR induction [9].

### 3.4. General Remarks

The strategy outlined in this study demonstrated the potential of transforming widely used biologically active molecules into their ionic derivatives, yielding bifunctional salts with tunable physicochemical and biological characteristics. Such an approach represents a promising pathway for the rational design of novel bioactive agents for sustainable agriculture [51,52]. The present work focuses on the preliminary screening of these newly synthesized compounds, while it is evident that the most promising candidates require further evaluation, both with respect to their safety of application and their effectiveness across a broader range of crop species and associated pathogens.

The next stage in the development of technology based on the use of SAR inducers requires the determination of an appropriate concentration of the selected promising candidate. In this context, two aspects must be taken into account. Firstly, the optimal concentration may vary depending on the crop species. Secondly, application of an excessive concentration of an SAR inducer may result in the occurrence of a phenomenon called growth–immunity trade-off [53,54]. Salicylic acid, which is involved in the regulation of numerous physiological processes in plants, including the induction of SAR, is also involved in the regulation of this phenomenon [55,56]. The shift of plant resources toward defense mechanisms can consequently lead to yield reduction. Therefore, the challenge lies in developing an application strategy that ensures the use of SAR inducers at concentrations and frequencies of treatment appropriate for a given crop.

In one of our previous studies, two newly synthesized derivatives belonging to another well-known group of SAR inducers, namely benzothiadiazoles, were tested in the field cultivation of tomato [57]. In contrast to salicylic acid and nicotinic acid derivatives, the compounds from the benzothiadiazole group are typically applied at much lower concentrations, around 20 mg/L. In our experiment, tomato plants were treated with two novel derivatives that were also obtained by our group, at concentrations of either 20 mg/L or 40 mg/L, with either six or nine applications performed during the growing season. The results indicated that for the treatment variants with the highest cumulative dose of SAR inducer, i.e., the compound used at a concentration of 40 mg/L applied nine times, the values of qualitative yield parameters were comparable to those obtained for the variant of standard fungicide treatment, which served as a reference. The variants of treatment tested in this study were intentionally selected to test whether the growth–immunity trade-off would occur and, if so, to what extent. For the remaining variants, in which the cumulative amount of SAR inducer applied was lower, a significant increase in the values of qualitative parameters of yield was observed compared to those obtained after the application of standard fungicide treatment. The number of treatments performed in this experiment should not be treated as optimal, but rather as a valuable reference point for the design of treatment variants to be tested in subsequent experiments. The general aim of future field studies will be to reduce the number of treatments performed and to strive to reduce the concentration of the SAR inducer used, which will also be important in the context of the cost-effectiveness of a given treatment variant. With such a technology developed for a given crop, it will be possible to test new active substances, which are indicated as compounds with high effectiveness in inducing SAR in screening tests such as those presented in this paper.

Should the safety issues related to the use of novel compounds in ionic form be of concern, the following conclusions from our other works should be noted. Our previous investigations demonstrated that the newly synthesized ionic derivatives were absorbed by plants to a significantly lower extent compared to the commercial fungicide formulated with the same active substance prior to derivatization. These findings indicate that, despite their ionic liquid nature, the derivatives exhibited improved safety, as they accumulated in plant tissues to a lesser degree than the neutral parent compound [58].

## 4. Materials and Methods

All reactions were carried out using commercially available solvents and reagents from Merck (Darmstadt, Germany), POCh S.A. (Gliwice, Poland), and CHEMPUR (Piekary Śląskie, Poland), without further purification unless otherwise stated. Salicylic acid and its chlorinated derivatives, nicotinic acid, isonicotinic acid, and chlormequat chloride, were used as received.

### 4.1. Synthesis of Potassium Salts of SAR Inducers

Potassium salts of the selected carboxylic acids were prepared by neutralizing 0.016 mol of each acid with 0.016 mol of KOH in distilled water (50 mL). The reaction mixture was stirred until dissolution was complete, followed by solvent evaporation (20 mbar, 60 °C) and drying under reduced pressure (24 h, 40 °C, 0.01 mbar). The resulting white crystalline potassium salts were used directly in the next step of the study.

### 4.2. Synthesis of Chlormequat-Based ILs

Equimolar amounts (0.01 mol) of 2-chloroethyltrimethylammonium chloride and the potassium salt of the SAR-inducing acid were dissolved separately in methanol (30 mL each). The two solutions were combined and stirred at room temperature for 8 h. After cooling to 4 °C, the mixture was filtered to remove precipitated KCl. The solvent was evaporated under reduced pressure (20 mbar, 40 °C), and the residue was dissolved in cold acetone to further remove residual inorganic impurities. After the second filtration of KCl and evaporation of acetone (40 mbar, 40 °C), the final products were dried under reduced pressure (24–48 h, 40 °C, 0.01 mbar).

### 4.3. Purification and Characterization

Halide contamination was assessed using an acidified AgNO_3_ solution, and chloride content was confirmed to be below 500 ppm. The purity and identity of the synthesized salts were verified by ^1^H and ^13^C NMR spectroscopy using Bruker spectrometers (400 MHz for ^1^H, 100 MHz for ^13^C), and by elemental analysis (AB Sciex TripleTOF^®^ 5600+ System with SCIEX Analyst TF 1.7 software, Framingham, MA, USA). Spectra were recorded in DMSO-d_6_ and referenced to residual solvent peaks.

### 4.4. Thermal Analysis

Melting points and glass transition temperatures were determined by DSC using the STARe System (Mettler Toledo, Greifensee, Switzerland). Samples (6–15 mg) were placed in sealed aluminum pans and subjected to a heating cycle from 25 °C to 160 °C at 10 °C/min, held isothermally, cooled to -80 °C, and reheated. TGA was performed on a TGA Q50 (Texas Instruments, Dallas, TX, USA) under nitrogen. Samples (5–10 mg) were heated from 25 °C to 500 °C at 10 °C/min with an isothermal step at 85 °C for 10 min. T_5%onset_ and T_onset_ values were recorded.

### 4.5. Solubility Tests

The solubilities of the prepared ILs in ten representative solvents were determined according to the protocols from Vogel’s Textbook of Practical Organic Chemistry [59]. The solvents chosen for the study were arranged in order of descending value of their Snyder polarity index: water—9.0, methanol—6.6, acetone—5.1, isopropanol—4.3, toluene—2.3, and hexane—0.0. A 0.1 g sample of each IL was added to a certain volume of a selected solvent and the samples were thermostated in a Huber D 77656 bath (Huber, Offenburg, Germany) at 25 °C. Based on the volume of solvent used, 3 types of solubility were recorded: ‘soluble’ applies to the compounds which dissolved in 1 mL of the solvent, ‘of limited solubility’ applies to those that dissolved in 3 mL of solvent, and ‘insoluble’ applies to those which did not dissolve in 3 mL of solvent.

### 4.6. Phytotoxicity Tests

The experiment was conducted under greenhouse conditions at 25 °C and 50% relative humidity, under natural daylight during the May–August growing season, with a natural photoperiod. Twenty five seeds of radish (*Raphanus sativus*) were placed on a filter tissue in a Petri dish. Filter tissue was moistened with 10 mL of water/glycol propylene (95/5 *v*/*v*) containing the tested active substance at a concentration of either 125 mg/L or 250 mg/L. Seeds were left to grow for 5 days at room temperature with access to sunlight. Then, sprouts were weighted. All variants of treatment were tested together, with 3 replications (plates) in each of 2 cycles performed. Results were subjected to statistical analysis as described in Section 4.8.

### 4.7. SAR Induction Assay

#### 4.7.1. Plants and Virus Materials

Plants of *Nicotiana tabacum* cv. Xanthi were grown under greenhouse conditions at 25 °C and 50% relative humidity, under natural daylight during the May–August growing season, with a natural photoperiod. The plants were sprayed with solutions containing the tested compounds.

In all experiments, the TMV SL-1 isolate Tobamovirus (TMV) was used. To obtain a viral inoculum, *Nicotiana tabacum* cv. Samsun plants were sap-inoculated with previously virus-infected plant material. After the appearance of disease symptoms on the infected plants (leaf mosaic), the virus was isolated by sucrose density gradient centrifugation with 0.01 M Tris-HCl, pH 7.0, to prepare the infectious inoculum [60]. The concentration of virus in the obtained preparation was determined spectrophotometrically. The samples of TMV were stored at −20 °C until use.

#### 4.7.2. Induction of Systemic Acquired Resistance

The tested active substances at a given concentration were sprayed on tobacco plants with three fully developed leaves. The volume of working solution used was equal to 10 mL per plant. The compounds were dissolved in a water/propylene glycol solution (95/5, *v*/*v*). The plants of the untreated control variant were sprayed with a water/propylene glycol solution (95/5, *v*/*v*) that was used for the preparation of solutions of the tested compounds. After seven days, the plants were mechanically inoculated using purified viral preparations (0.01 ng/mL as described in [34]).

After a week, the area of necrotic spots covering the leaves of the plants treated with the tested active substances was calculated and compared to that of the leaves from untreated control plants. The plants were inoculated with the TMV inoculum at a concentration of 3.8 × 10^−4^ ng per leaf. For each variant of treatment, 3 plants were sprayed, and 3 leaves of each plant were analyzed. The entire experiment was performed in 2 replicates. Necrotic areas on the leaves were determined using ImageJ software [61].

Reduction in the area of necrotic spots on the leaves treated with the tested compounds, in comparison to that appearing on the untreated plants, shows inhibition of viral infection by induction of plant resistance through the use of the tested compounds. The surface of a leaf or an area with necrotic spots is represented by a set of pixels in the image, which are counted by the program. Reduction in necrotic area (I_SAR_) is expressed by the following formula:

(1) $$I_{SAR}=100-(\frac{At}{Ac}*100)$$

where *At* is the ratio of the area of necrotic spots caused by viral infection that appeared on the leaf treated with the test substance to the whole area of the leaf; *Ac* is the ratio of the area of the necrotic spots caused by viral infection that appeared on the leaf of the control plant to the whole area of the leaf.

### 4.8. Statistical Analysis

Statistical significance of the effect of the treatments of plants with tested compounds on the phytotoxicity and SAR induction was assessed with the ANOVA test performed using the Statistica program StatSoft with software version 9.1 (StatSoft Sp. z o.o., Kraków, Poland). The statistical assumptions of normality and homogeneity of variances were verified prior to conducting the ANOVA tests. Differences between the means were estimated with Tukey’s HSD test at the significance level of *p* = 0.05.

## Figures and Tables

**Table 1:** Thermal properties of the obtained chlormequat-based ILs.

No	Compound Abbreviation	Thermal Stability T_5%onset_ [°C]	Thermal Stability T_onset_ [°C]	Melting Point T_peak_ [°C]	Glass Transition T_peak_ [°C]
**1.0**	[CC][Cl]	255.0	240 *	240 *	-
**1.1**	[CC][3-ClSal]	196.2	136.1	136.1	1.3
**1.2**	[CC][4-ClSal]	205.3	137.2	137.2	4.7
**1.3**	[CC][5-ClSal]	217.1	138.5	138.5	5.1
**1.4**	[CC][3,5-ClSal]	208.3	140.1	140.1	7.3
**1.5**	[CC][Sal]	186.1	93.3	93.3	13.8
**1.6**	[CC][IsoNic]	164.3	85.3	85.3	
**1.7**	[CC][Ina]	177.2	70.3	70.3	30.6
**1.8**	[CC][Nic]	166.9	89.9	89.9	32.2

*—melting point with simultaneous decay.

**Table 2:** Solubility of chlormequat-based ILs at 25 °C.

No	Compound Abbreviation	Water	Methanol	Acetone	Isopropanol	Toluene	Hexane
Snyder polarity index		9.0	6.6	5.1	4.3	2.3	0.0
**1.0**	[CC][Cl]	+	+	−	−	−	−
**1.1**	[CC][3-ClSal]	+/−	+	−	+/−	−	−
**1.2**	[CC][4-ClSal]	+/−	+	−	+/−	−	−
**1.3**	[CC][5-ClSal]	+/−	+	−	+/−	−	−
**1.4**	[CC][3,5-ClSal]	+/−	+	−	+/−	−	−
**1.5**	[CC][Sal]	+	+	−	+/−	−	−
**1.6**	[CC][IsoNic]	+	+	−	+/−	−	−
**1.7**	[CC][Ina]	+	+	−	+/−	−	−
**1.8**	[CC][Nic]	+	+	−	+/−	−	−

+ soluble; +/− of limited solubility; − insoluble.

**Table 3:** Masses of 25 radish sprouts resulting from the application of the tested variants of treatment consisting of chlormequat-based ILs and their corresponding carboxylic acids.

	Variant of Treatment	Mass of 25 Sprouts [g]	
		125 mg/L	250 mg/L
UTC		3.09 i C	3.09 k C
[CC][Cl]		2.35 def B	2.08 fgh A
**1.1**	[CC][3-ClSal]	2.60 fgh D	1.96 ef C
	3-chlorosalicylic acid	1.64 a B	0.85 a A
**1.2**	[CC][4-ClSal]	2.84 hi D	2.48 ij C
	4-chlorosalicylic acid	1.65 a B	0.98 ab A
**1.3**	[CC][5-ClSal]	2.50 efg D	2.16 fgh C
	5-chlorosalicylic acid	1.94 abc B	1.24 cd A
**1.4**	[CC][3,5-ClSal]	2.24 cde C	1.94 ef B
	3,5-dichlorosalicylic acid	2.07 bcd B	1.25 cd A
**1.5**	[CC][Sal]	2.82 hi C	2.26 hi B
	Salicylic acid	2.17 cd B	1.14 bc A
**1.6**	[CC][IsoNic]	2.60 fgh C	1.99 efg AB
	Isonicotinic acid	2.19 cde B	1.77 e A
**1.7**	[CC][Ina]	2.63 fgh D	2.22 gh C
	2,6-dichloroisonicotinic acid	1.85 ab B	1.41 d A
**1.8**	[CC][Nic]	2.84 hi B	2.28 hi A
	Nicotinic acid	2.76 gh B	2.63 j AB

Symbols for the table: for the pair of rows referring to the compound in the form of acid and its salt derivative, mean values marked with the same capital letters do not differ significantly at *p* = 0.05; for the columns separately, mean values marked with the same small letters do not differ significantly at *p* = 0.05.

**Table 4:** Percentage reduction in the masses of sprouts treated with chlormequat-based ionic liquids and the corresponding carboxylic acids compared with untreated control plants. Compounds are listed in descending order of efficiency for each tested concentration.

	Tested Compound in Concentration of 125 mg/L	Reduction in Sprout Mass [%]	Tested Compound in Concentration of 250 mg/L	Reduction in Sprout Mass [%]
1	3-chlorosalicylic acid	47.03	3-chlorosalicylic acid	72.65
2	4-chlorosalicylic acid	46.55	4-chlorosalicylic acid	68.45
3	2,6-dichloroisonicotinic acid	40.29	SAL	63.27
4	5-chlorosalicylic acid	37.16	5-chlorosalicylic acid	59.87
5	3,5-dichlorosalicylic acid	32.96	3,5-dichlorosalicylic acid	59.49
6	SAL	29.77	2,6-dichloroisonicotinic acid	54.31
7	Isonicotinic acid	29.07	Isonicotinic acid	42.83
8	[CC][3,5-ClSal]	27.51	[CC][3,5-ClSal]	37.38
9	[CC][Cl]	24.00	[CC][3-ClSal]	36.68
10	[CC][5-ClSal]	19.15	[CC][IsoNic]	35.65
11	[CC][3-ClSal]	15.97	[CC][Cl]	32.69
12	[CC][IsoNic]	15.70	[CC][5-ClSal]	30.15
13	[CC][Ina]	15.05	[CC][Ina]	28.21
14	Nicotinic acid	10.79	[CC][Sal]	26.91
15	[CC][Sal]	8.79	[CC][Nic]	26.16
16	[CC][Nic]	8.20	[CC][4-ClSal]	19.63

**Table 5:** Systemic acquired resistance (SAR)-inducing properties of the obtained chlormequat-based ILs compared with the untreated control and the corresponding carboxylic acid with SAR-inducing activity.

Variant of Treatment		SAR Induction [%]	
		125 mg/L	250 mg/L
UTC		34.54 h	34.54 h
**1.1**	[CC][3-ClSal]	2.52 a AB	1.39 a A
	3-chlorosalicylic acid	4.95 a B	2.59 a AB
**1.2**	[CC][4-ClSal]	12.47 bc B	7.85 bcde A
	4-chlorosalicylic acid	17.06 cd C	8.68 cde AB
**1.3**	[CC][5-ClSal]	3.15 a AB	1.58 a A
	5-chlorosalicylic acid	4.23 a B	3.28 ab AB
**1.4**	[CC][3,5-ClSal]	7.79 ab A	4.93 abc A
	3,5-dichlorosalicylic acid	15.03 cd B	7.51 bcd A
**1.5**	[CC][Sal]	19.99 de B	12.39 ef A
	Salicylic acid	26.60 fg C	25.06 g C
**1.6**	[CC][IsoNic]	24.51 ef A	21.48 g A
	Isonicotinic acid	31.12 gh B	25.58 g A
**1.7**	[CC][Ina]	14.80 cd AB	12.52 ef A
	2,6-dichloroisonicotinic acid	18.15 cd B	14.90 f AB
**1.8**	[CC][Nic]	19.70 de B	11.52 def A
	Nicotinic acid	27.69 fg C	22.07 g B

Symbols for the table: for the pair of rows referring to the compound in the form of acid and its salt derivative, mean values marked with the same capital letters do not differ significantly at *p* = 0.05; for the columns separately, mean values marked with the same small letters do not differ significantly at *p* = 0.05.

**Table 6:** Reduction (%) in necrotic leaf area in plants treated with chlormequat-based ionic liquids and the corresponding carboxylic acids with known SAR-inducing activity, compared with untreated control plants. Compounds are listed in descending order of efficiency for each tested concentration (125 and 250 mg/L).

Tested Compound in Concentration of 125 mg/L		Reduction in Necrotic Area [%]	Tested Compound in Concentration of 250 mg/L	Reduction in Necrotic Area [%]
1	[CC][3-ClSal]	92.7	[CC][3-ClSal]	96.0
2	[CC][5-ClSal]	90.9	[CC][5-ClSal]	95.4
3	5-chlorosalicylic acid	87.8	3-chlorosalicylic acid	92.5
4	3-chlorosalicylic acid	85.7	5-chlorosalicylic acid	90.5
5	[CC][3,5-ClSal]	77.4	[CC][3,5-ClSal]	85.7
6	[CC][4-ClSal]	63.9	3,5-dichlorosalicylic acid	78.3
7	[CC][Ina]	57.2	[CC][4-ClSal]	77.3
8	3,5-dichlorosalicylic acid	56.5	4-chlorosalicylic acid	74.9
9	4-chlorosalicylic acid	50.6	[CC][Nic]	66.7
10	2,6-dichloroisonicotinic acid	47.5	[CC][Sal]	64.1
11	[CC][Nic]	43.0	[CC][Ina]	63.8
12	[CC][Sal]	42.1	2,6-dichloroisonicotinic acid	56.9
13	[CC][Isonicl]	29.0	[CC][Isonicl]	37.8
14	Salicylic acid	23.0	Nicotinic acid	36.1
15	Nicotinic acid	19.8	Salicylic acid	27.4
16	Isonicotinic acid	9.9	Isonicotinic acid	26.0

## Data Availability

The original contributions presented in this study are included in the Supplementary Materials. Further inquiries can be directed to the corresponding author.

## Supplementary Materials

The following supporting information can be downloaded at https://www.mdpi.com/article/10.3390/molecules30214203/s1: Table S1: Raw data for calculation of systemic acquired resistance (SAR) inducing properties of the obtained chlormequat-based ILs. Figure S1: Induction of SAR triggered by spraying with [CC][3-ClSal] at a concentration of 250 mg/L. 1—total leaf area; 2—area of necrotic spots. Percentage of necrotic spot coverage = 0.27%.; Figure S2: Induction of SAR triggered by spraying with [CC][5-ClSal] at a concentration of 250 mg/L. 1—total leaf area; 2—area of necrotic spots. Percentage of necrotic spot coverage = 1.15%; Figure S3: Induction of SAR triggered by spraying with 3-chlorosalicylic acid at a concentration of 250 mg/L. 1—total leaf area; 2—area of necrotic spots. Percentage of necrotic spot coverage = 2.30%; Figure S4: Induction of SAR triggered by spraying with 5-chlorosalicylic acid at a concentration of 250 mg/L. 1—total leaf area; 2—area of necrotic spots. Percentage of necrotic spot coverage = 1.28%; Figure S5: Induction of SAR triggered by spraying with salicylic acid at a concentration of 250 mg/L. 1—total leaf area; 2—area of necrotic spots. Percentage of necrotic spot coverage = 19.86%; Figure S6: Induction of SAR triggered by spraying with [CC][3-ClSal] at a concentration of 125 mg/L. 1—total leaf area; 2—area of necrotic spots. Percentage of necrotic spot coverage = 0.79%; Figure S7: Induction of SAR triggered by spraying with [CC][5-ClSal] at a concentration of 125 mg/L. 1—total leaf area; 2—area of necrotic spots. Percentage of necrotic spot coverage = 2.28%; Figure S8: Induction of SAR triggered by spraying with 3-chlorosalicylic acid at a concentration of 125 mg/L. 1—total leaf area; 2—area of necrotic spots. Percentage of necrotic spot coverage = 2.62%; Figure S9: Induction of SAR triggered by spraying with 5-chlorosalicylic acid at a concentration of 125 mg/L. 1—total leaf area; 2—area of necrotic spots. Percentage of necrotic spot coverage = 2.98%; Figure S10: Induction of SAR triggered by spraying with salicylic acid at a concentration of 125 mg/L. 1—total leaf area; 2—area of necrotic spots. Percentage of necrotic spot coverage = 23.67%; Figure S11: No induction of SAR effect—plants sprayed only with water (UTC). 1—total leaf area; 2—area of necrotic spots. Percentage of necrotic spot coverage = 38.44%.
