# Supplementary material for: New Chlormequat-Based Ionic Liquids as Plant Resistance Inducers
Source: Molecules. 2025 Oct 27;30(21):4203. doi: 10.3390/molecules30214203 (PMC12610573; doi:10.3390/molecules30214203)

ESI

New Chlormequat-Based Ionic Liquids as Plant Resistance Inducers

Rafal Kukawka [1,2,*], Maciej Spychalski [1,2], Patrycja Czerwoniec [1,3], Beata Hasiów-Jaroszewska [4], Sylwia Stępniewska-Jarosz [4], Emilia Frydrych-Tomczak [1] and Marcin Smiglak [1]

[1] Poznan Science and Technology Park, Adam Mickiewicz University Foundation, Ul. Rubiez 46, 61-612 Poznań, Poland;
[2] ATI Sp. z o.o., Ul. Rubież 46, 61-612 Poznań, Poland
[3] Faculty of Chemistry, Adam Mickiewicz University, Ul. Uniwersytetu Poznańskiego 8, 61-614 Poznań, Poland
[4] Institute of Plant Protection—National Research Institute, Ul. Wł. Węgorka 20, 60-318 Poznań, Poland
* Correspondence: rafal.kukawka@ppnt.poznan.pl

Pages: 4

Table: 1

Figures: 11

Table of content

^{1}H, ^{13}C NMR, HRMS data of obtained chlormequat-based ILs……………………………..……………3

Raw data for calculation of systemic acquired resistance (SAR) inducing properties of the obtained chlormequat-based ILs……………………………………………………………………………………5

Examples of leaves treated with chlormequat-based ILs: total leaf area; area of necrotic spots and percentage of necrotic spot coverage…………………………………………………………………….26

^{1}H, ^{13}C NMR, HRMS data of obtained chlormequat-based ILs

2-chloro-*N*,*N*,*N*-trimethylethan-1-aminium 3-chlorosalicylate, [CC][3-ClSal] (1.1)

Yield 90%, white crystalline solid.

^{1}H NMR (400 MHz, DMSO-*d*$_6$) δ 8.46 (dd, *J* = 4.7, 1.9 Hz, 1H), 8.12 (dd, *J* = 7.5, 1.6 Hz, 1H), 7.40 (dd, *J* = 7.6, 4.8 Hz, 1H), 4.13 (t, *J* = 6.8 Hz, 2H), 3.79 (t, *J* = 6.9 Hz, 2H), 3.19 (s, 9H).

^{13}C NMR (101 MHz, DMSO-*d*$_6$) δ 167.0, 151.9, 135.0, 129.6, 124.3, 123.8, 119.8, 65.4, 53.6, 36.7.

HRMS m/z: Calcd for $C_5H_{13}ClN^+$ 122.0731; Found 122.0734,

HRMS m/z: Calcd for $C_7H_4ClO_3^-$ 170.9849; Found 170.9854.

2-chloro-*N*,*N*,*N*-trimethylethan-1-aminium 4-chlorosalicylate, [CC][4-ClSal] (1.2)

Yield 92%, white crystalline solid.

^{1}H NMR (400 MHz, DMSO-*d*$_6$) δ 7.79 (d, 1H), 7.07, 6.89 (d, 1H), 4.09 (t, *J* = 6.8 Hz, 2H), 3.82 (t, *J* = 6.9 Hz, 2H), 3.18 (s, 9H).

^{13}C NMR (101 MHz, DMSO-*d*$_6$) δ 171.0, 160.9, 139.0, 131.6, 124.8, 121.2, 119.3, 65.2, 54.1, 37.1.

HRMS m/z: Calcd for $C_5H_{13}ClN^+$ 122.0731; Found 122.0730,

HRMS m/z: Calcd for $C_7H_4ClO_3^-$ 170.9849; Found 170.9852.

2-chloro-*N*,*N*,*N*-trimethylethan-1-aminium 5-chlorosalicylate, [CC][5-ClSal] (1.3)

Yield 92%, white crystalline solid.

^{1}H NMR (400 MHz, DMSO-*d*$_6$) δ 7.61 (d, *J* = 2.9 Hz, 1H), 7.16 (dd, *J* = 8.6, 2.9 Hz, 1H), 6.67 (d, *J* = 8.6 Hz, 1H), 4.11 (t, *J* = 6.8 Hz, 2H), 3.77 (t, *J* = 6.9 Hz, 2H), 3.16 (s, 9H).

^{13}C NMR (101 MHz, DMSO-*d*$_6$) δ 173.8, 167.8, 136.1, 133.4, 129.5, 123.9, 118.8, 65.3, 53.4, 36.6.

HRMS m/z: Calcd for $C_5H_{13}ClN^+$ 122.0731; Found 122.0733,

HRMS m/z: Calcd for $C_7H_4ClO_3^-$ 170.9849; Found 170.9853.

2-chloro-*N*,*N*,*N*-trimethylethan-1-aminium 3,5-chlorosalicylate, [CC][3,5-ClSal] (1.4)

Yield 93%, white crystalline solid.

^{1}H NMR (400 MHz, DMSO-*d*$_6$) δ 7.54 (d, *J* = 2.8 Hz, 1H), 7.36 (d, *J* = 2.8 Hz, 1H), 4.13 (t, *J* = 6.8 Hz, 2H), 3.80(t, *J* = 6.9 Hz, 2H), 3.15 (s, 9H).

^{13}C NMR (101 MHz, DMSO-*d*$_6$) δ 169.7, 159.9, 131.5, 128.2, 121.9, 121.4, 118.4, 65.5, 53.2, 36.5.

HRMS m/z: Calcd for $C_5H_{13}ClN^+$ 122.0731; Found 122.0732,

HRMS m/z: Calcd for $C_7H_3Cl_2O_3^-$ 204.9465; Found 204.9466.

2-chloro-*N*,*N*,*N*-trimethylethan-1-aminium salicylate, [CC][Sal] (1.5)

Yield 95%, red wax.

^{1}H NMR (400 MHz, DMSO-*d*$_6$) δ 7.66 (d, *J* = 7.6 Hz, 1H), 7.12 (t, *J* = 7.6 Hz, 1H), 6.59 (dd, *J* = 14.6, 7.6 Hz, 2H), 4.11 (t, *J* = 6.8 Hz, 2H), 3.78 (t, *J* = 6.6 Hz, 2H), 3.16 (s, 9H).

^{13}C NMR (101 MHz, DMSO-*d*$_6$) δ 172.1, 163.2, 132.0, 130.4, 120.5, 116.6, 116.3, 65.2, 53.1, 36.9.

HRMS m/z: Calcd for $C_5H_{13}ClN^+$ 122.0731; Found 122.0733,
HRMS m/z: Calcd for $C_7H_5O_3^-$ 137.0239; Found 137.0237.

2-chloro-*N,N,N*-trimethylethan-1-aminium isonicotinate, [CC][Isonic] (1.6)

Yield 92%, white wax.
^{1}H NMR (400 MHz, DMSO-*d*$_6$) δ 9.21 (d, 2H), 8.58 (d, 2H), 4.12 (t, *J* = 6.4 Hz, 2H), 3.83 (t, *J* = 6.5 Hz, 2H), 3.11 (s, 9H).
^{13}C NMR (101 MHz, DMSO-*d*$_6$) δ 166.5.0, 151.3, 137.4, 123.5, 65.6, 53.4, 37.3.
HRMS m/z: Calcd for $C_5H_{13}ClN^+$ 122.0731; Found 122.0734,
HRMS m/z: Calcd for $C_6H_4NO_2^-$ 122.0247; Found 122.0249.

2-chloro-*N,N,N*-trimethylethan-1-aminium 2,6-dichloroisonicotinate, [CC][Ina] (1.7)

Yield 96%, white wax.
^{1}H NMR (400 MHz, DMSO-*d*$_6$) δ 7.66 (s, 2H), 4.11 (t, *J* = 6.8 Hz, 2H), 3.77 (t, *J* = 6.9 Hz, 2H), 3.15 (s, 9H).
^{13}C NMR (101 MHz, DMSO-*d*$_6$) δ167.0, 157.2, 151.4, 124.9, 65.3, 53.6, 37.1.
HRMS m/z: Calcd for C5H13ClN$^+$ 122.0731; Found 122.0734
HRMS m/z: Calcd for $C_6H_2Cl_2NO_2^-$ 189.9463; Found 189.9467.

2-chloro-*N,N,N*-trimethylethan-1-aminium nicotinate, [CC][Nic] (1.8)

Yield 92%, white wax.
^{1}H NMR (400 MHz, DMSO-*d*$_6$) δ 9.21 (s, 1H), 8.97 (d, *J* = 4.1 Hz, 1H), 8.38 (d, *J* = 7.9 Hz, 1H), 7.52 (dd, *J* = 7.9, 5.1 Hz, 1H), 4.13 (t, *J* = 6.4 Hz, 2H), 3.85 (t, *J* = 6.5 Hz, 2H), 3.22 (s, 9H).
^{13}C NMR (101 MHz, DMSO-*d*$_6$) δ 175.0, 155.3, 152.2, 136.9, 131.2, 123.2, 65.6, 53.5, 37.2.
HRMS m/z: Calcd for $C_5H_{13}ClN^+$ 122.0731; Found 122.0734,
HRMS m/z: Calcd for $C_6H_4NO_2^-$ 122.0247; Found 122.0245.

Raw data for calculation of systemic acquired resistance (SAR) inducing properties of the obtained chlormequat-based ILs.

Table S1. Raw data for calculation **of** systemic acquired resistance (SAR) inducing properties of the obtained chlormequat-based ILs.

Number	Compound	Concentration [mg/L]	Experminet number	Plant Number	Leaf number	Treatment code	Total leaf area [pixels^2]	Area covered by necrosis [pixels^2]	Percentage of leaf infection [%]	Standard deviation of percentage of leaf infection	Remarks
1	[CC][2,6-diClIsonic]	250	1	1	1	250z1	1045321	95960	9.18		
1	[CC][2,6-diClIsonic]	250	1	1	2	250z1	952314	88280	9.27		
1	[CC][2,6-diClIsonic]	250	1	1	3	250z1	803221	81286	10.12		
1	[CC][2,6-diClIsonic]	250	1	1	1	250z1	1103456	112442	10.19		
1	[CC][2,6-diClIsonic]	250	1	1	2	250z1	971025	125456	12.92		
1	[CC][2,6-diClIsonic]	250	1	1	3	250z1	829115	124865	15.06		
1	[CC][2,6-diClIsonic]	250	1	2	1	250z1	1011789	153893	15.21		
1	[CC][2,6-diClIsonic]	250	1	2	2	250z1	939876	143331	15.25		
1	[CC][2,6-diClIsonic]	250	1	2	3	250z1	775904	119877	15.45		
1	[CC][2,6-diClIsonic]	250	2	2	1	250z1	1085432	99643	9.18		
1	[CC][2,6-diClIsonic]	250	2	2	2	250z1	965110	89466	9.27		
1	[CC][2,6-diClIsonic]	250	2	2	3	250z1	836420	84646	10.12		
1	[CC][2,6-diClIsonic]	250	2	3	1	250z1	1064210	108443	10.19		
1	[CC][2,6-diClIsonic]	250	2	3	2	250z1	924558	119453	12.92		
1	[CC][2,6-diClIsonic]	250	2	3	3	250z1	762559	114841	15.06		
1	[CC][2,6-diClIsonic]	250	2	3	1	250z1	1097654	166953	15.21		
1	[CC][2,6-diClIsonic]	250	2	3	2	250z1	978443	149213	15.25		
1	[CC][2,6-diClIsonic]	250	2	3	3	250z1	815337	125970	15.45	2.72	
2	2,6-dichloroisonicotinic acid	250	1	1	1	250z2	1023875	120817	11.80		
2	2,6-dichloroisonicotinic acid	250	1	1	2	250z2	909772	110628	12.16		

2	2,6-dichloroisonicotinic acid	250	1	1	3	250z2	788906	98534	12.49		
2	2,6-dichloroisonicotinic acid	250	1	1	1	250z2	1111230	140682	12.66		
2	2,6-dichloroisonicotinic acid	250	1	1	2	250z2	956680	140345	14.67		
2	2,6-dichloroisonicotinic acid	250	1	1	3	250z2	833770	140407	16.84		
2	2,6-dichloroisonicotinic acid	250	1	2	1	250z2	1008942	174850	17.33		
2	2,6-dichloroisonicotinic acid	250	1	2	2	250z2	983210	173340	17.63		
2	2,6-dichloroisonicotinic acid	250	1	2	3	250z2	754118	139738	18.53		
2	2,6-dichloroisonicotinic acid	250	2	2	1	250z2	1056778	124700	11.80		
2	2,6-dichloroisonicotinic acid	250	2	2	2	250z2	948125	115292	12.16		
2	2,6-dichloroisonicotinic acid	250	2	2	3	250z2	838660	104749	12.49		
2	2,6-dichloroisonicotinic acid	250	2	3	1	250z2	1072334	135757	12.66		
2	2,6-dichloroisonicotinic acid	250	2	3	2	250z2	967333	141908	14.67		
2	2,6-dichloroisonicotinic acid	250	2	3	3	250z2	769305	129551	16.84		
2	2,6-dichloroisonicotinic acid	250	2	3	1	250z2	1094501	189677	17.33		
2	2,6-dichloroisonicotinic acid	250	2	3	2	250z2	934209	164701	17.63		
2	2,6-dichloroisonicotinic acid	250	2	3	3	250z2	821499	152224	18.53	2.62	
**3**	**[CC][3-ClSal]**	**250**	**1**	**1**	**1**	**250z3**	**873725**	**2395**	**0.27**		**Fig. S1**
3	[CC][3-ClSal]	250	1	1	2	250z3	975876	488	0.05		
3	[CC][3-ClSal]	250	1	1	3	250z3	779882	3198	0.41		
3	[CC][3-ClSal]	250	1	1	1	250z3	1086915	10978	1.01		
3	[CC][3-ClSal]	250	1	1	2	250z3	943510	0	0.00		
3	[CC][3-ClSal]	250	1	1	3	250z3	807431	1453	0.18		
3	[CC][3-ClSal]	250	1	2	1	250z3	1031448	15472	1.50		
3	[CC][3-ClSal]	250	1	2	2	250z3	958792	41995	4.38		
3	[CC][3-ClSal]	250	1	2	3	250z3	835266	39174	4.69		
3	[CC][3-ClSal]	250	2	2	1	250z3	1076223	3767	0.35		
3	[CC][3-ClSal]	250	2	2	2	250z3	969401	485	0.05		
3	[CC][3-ClSal]	250	2	2	3	250z3	758944	3112	0.41		
3	[CC][3-ClSal]	250	2	3	1	250z3	1108377	11195	1.01		
3	[CC][3-ClSal]	250	2	3	2	250z3	928665	0	0.00		

3	[CC][3-ClSal]	250	2	3	3	250z3	828120	828	0.10		
3	[CC][3-ClSal]	250	2	3	1	250z3	1002519	15038	1.50		
3	[CC][3-ClSal]	250	2	3	2	250z3	982044	43014	4.38		
3	[CC][3-ClSal]	250	2	3	3	250z3	773690	36286	4.69	1.80	
4	3-chlorosalicylic acid	250	1	1	1	250z4	1101588	1763	0.16		
4	3-chlorosalicylic acid	250	1	1	2	250z4	872665	2094	0.24		
4	3-chlorosalicylic acid	250	1	1	3	250z4	814577	5783	0.71		
4	3-chlorosalicylic acid	250	1	1	1	250z4	1152695	8299	0.72		
4	3-chlorosalicylic acid	250	1	1	2	250z4	936542	18544	1.98		
4	3-chlorosalicylic acid	250	1	1	3	250z4	785563	35900	4.57		
4	3-chlorosalicylic acid	250	1	2	1	250z4	1022740	49194	4.81		
4	3-chlorosalicylic acid	250	1	2	2	250z4	926831	45971	4.96		
4	3-chlorosalicylic acid	250	1	2	3	250z4	879112	45626	5.19		
4	3-chlorosalicylic acid	250	2	2	1	250z4	944956	1512	0.16		
4	3-chlorosalicylic acid	250	2	2	2	250z4	910660	2186	0.24		
4	3-chlorosalicylic acid	250	2	2	3	250z4	758389	5385	0.71		
4	3-chlorosalicylic acid	250	2	3	1	250z4	1041124	7496	0.72		
**4**	**3-chlorosalicylic acid**	**250**	**2**	**3**	**2**	**250z4**	**919943**	**21178**	**2.30**		**Fig. S3**
4	3-chlorosalicylic acid	250	2	3	3	250z4	832883	38063	4.57		
4	3-chlorosalicylic acid	250	2	3	1	250z4	1098599	52843	4.81		
4	3-chlorosalicylic acid	250	2	3	2	250z4	959279	47580	4.96		
4	3-chlorosalicylic acid	250	2	3	3	250z4	800687	3899	4.87	2.15	
5	[CC][4-ClSal]	250	1	1	1	250z5	1090187	48077	4.41		
5	[CC][4-ClSal]	250	1	1	2	250z5	878334	46376	5.28		
5	[CC][4-ClSal]	250	1	1	3	250z5	788945	42287	5.36		
5	[CC][4-ClSal]	250	1	1	1	250z5	1029809	57772	5.61		
5	[CC][4-ClSal]	250	1	1	2	250z5	990000	75834	7.66		
5	[CC][4-ClSal]	250	1	1	3	250z5	820118	82012	10.00		
5	[CC][4-ClSal]	250	1	2	1	250z5	1098077	110467	10.06		
5	[CC][4-ClSal]	250	1	2	2	250z5	953981	97211	10.19		

5	[CC][4-ClSal]	250	1	2	3	250z5	805199	97590	12.12	
5	[CC][4-ClSal]	250	2	2	1	250z5	1016835	44842	4.41	
5	[CC][4-ClSal]	250	2	2	2	250z5	966448	51028	5.28	
5	[CC][4-ClSal]	250	2	2	3	250z5	800218	42892	5.36	
5	[CC][4-ClSal]	250	2	3	1	250z5	1047600	58770	5.61	
5	[CC][4-ClSal]	250	2	3	2	250z5	908819	69616	7.66	
5	[CC][4-ClSal]	250	2	3	3	250z5	806742	80674	10.00	
5	[CC][4-ClSal]	250	2	3	1	250z5	1034662	104087	10.06	
5	[CC][4-ClSal]	250	2	3	2	250z5	984685	100339	10.19	
5	[CC][4-ClSal]	250	2	3	3	250z5	789149	95645	12.12	2.72
6	4-chlorosalicylic acid	250	1	1	1	250z6	1159779	60193	5.19	
6	4-chlorosalicylic acid	250	1	1	2	250z6	1156132	62084	5.37	
6	4-chlorosalicylic acid	250	1	1	3	250z6	913622	50341	5.51	
6	4-chlorosalicylic acid	250	1	1	1	250z6	1107071	68860	6.22	
6	4-chlorosalicylic acid	250	1	1	2	250z6	999419	84151	8.42	
6	4-chlorosalicylic acid	250	1	1	3	250z6	704626	77227	10.96	
6	4-chlorosalicylic acid	250	1	2	1	250z6	1166178	137259	11.77	
6	4-chlorosalicylic acid	250	1	2	2	250z6	1104381	135397	12.26	
6	4-chlorosalicylic acid	250	1	2	3	250z6	831578	103698	12.47	
6	4-chlorosalicylic acid	250	2	2	1	250z6	1165032	60465	5.19	
6	4-chlorosalicylic acid	250	2	2	2	250z6	935148	50217	5.37	
6	4-chlorosalicylic acid	250	2	2	3	250z6	780009	42978	5.51	
6	4-chlorosalicylic acid	250	2	3	1	250z6	1003966	62447	6.22	
6	4-chlorosalicylic acid	250	2	3	2	250z6	953330	80270	8.42	
6	4-chlorosalicylic acid	250	2	3	3	250z6	834531	91465	10.96	
6	4-chlorosalicylic acid	250	2	3	1	250z6	1060638	124837	11.77	
6	4-chlorosalicylic acid	250	2	3	2	250z6	960723	117785	12.26	
6	4-chlorosalicylic acid	250	2	3	3	250z6	772779	96366	12.47	3.09
7	[CC][5-ClSal]	250	1	1	1	250z7	1088092	1197	0.11	
7	[CC][5-ClSal]	250	1	1	2	250z7	892576	2231	0.25	

7	[CC][5-ClSal]	250	1	1	3	250z7	787597	3072	0.39		
7	[CC][5-ClSal]	250	1	1	1	250z7	1071612	11038	1.03		
7	[CC][5-ClSal]	250	1	1	2	250z7	957355	287	0.03		
**7**	**[CC][5-ClSal]**	**250**	**1**	**1**	**3**	**250z7**	**827054**	**9532**	**1.15**		**Fig. S2**
7	[CC][5-ClSal]	250	1	2	1	250z7	1018892	24046	2.36		
7	[CC][5-ClSal]	250	1	2	2	250z7	940206	41933	4.46		
7	[CC][5-ClSal]	250	1	2	3	250z7	819911	38454	4.69		
7	[CC][5-ClSal]	250	2	2	1	250z7	1052269	1157	0.11		
7	[CC][5-ClSal]	250	2	2	2	250z7	929492	2324	0.25		
7	[CC][5-ClSal]	250	2	2	3	250z7	791178	3086	0.39		
7	[CC][5-ClSal]	250	2	3	1	250z7	1121655	11553	1.03		
7	[CC][5-ClSal]	250	2	3	2	250z7	959185	288	0.03		
7	[CC][5-ClSal]	250	2	3	3	250z7	793359	7537	0.95		
7	[CC][5-ClSal]	250	2	3	1	250z7	1047792	24728	2.36		
7	[CC][5-ClSal]	250	2	3	2	250z7	919014	40988	4.46		
7	[CC][5-ClSal]	250	2	3	3	250z7	807790	37885	4.69	1.78	
8	5-chlorosalicylic acid	250	1	1	1	250z8	965682	1062	0.11		
8	5-chlorosalicylic acid	250	1	1	2	250z8	820567	5334	0.65		
8	5-chlorosalicylic acid	250	1	1	3	250z8	657277	4535	0.69		
8	5-chlorosalicylic acid	250	1	1	1	250z8	991745	8430	0.85		
8	5-chlorosalicylic acid	250	1	1	2	250z8	927412	27730	2.99		
8	5-chlorosalicylic acid	250	1	1	3	250z8	812530	41927	5.16		
8	5-chlorosalicylic acid	250	1	2	1	250z8	927784	48245	5.20		
8	5-chlorosalicylic acid	250	1	2	2	250z8	914784	58821	6.43		
8	5-chlorosalicylic acid	250	1	2	3	250z8	659688	49081	7.44		
8	5-chlorosalicylic acid	250	2	2	1	250z8	1050305	1155	0.11		
8	5-chlorosalicylic acid	250	2	2	2	250z8	926406	6022	0.65		
8	5-chlorosalicylic acid	250	2	2	3	250z8	775161	5349	0.69		
**8**	**5-chlorosalicylic acid**	**250**	**2**	**3**	**1**	**250z8**	**813050**	**10422**	**1.28**		**Fig. S4**
8	5-chlorosalicylic acid	250	2	3	2	250z8	940413	28118	2.99		

8	5-chlorosalicylic acid	250	2	3	3	250z8	718065	37052	5.16	
8	5-chlorosalicylic acid	250	2	3	1	250z8	940556	48909	5.20	
8	5-chlorosalicylic acid	250	2	3	2	250z8	931196	59876	6.43	
8	5-chlorosalicylic acid	250	2	3	3	250z8	818458	60893	7.44	2.73
9	[CC][3,5-ClSal]	250	1	1	1	250z9	1110749	7442	0.67	
9	[CC][3,5-ClSal]	250	1	1	2	250z9	917183	15500	1.69	
9	[CC][3,5-ClSal]	250	1	1	3	250z9	778223	16654	2.14	
9	[CC][3,5-ClSal]	250	1	1	1	250z9	1100980	24222	2.20	
9	[CC][3,5-ClSal]	250	1	1	2	250z9	947351	49831	5.26	
9	[CC][3,5-ClSal]	250	1	1	3	250z9	782487	58843	7.52	
9	[CC][3,5-ClSal]	250	1	2	1	250z9	1036557	81162	7.83	
9	[CC][3,5-ClSal]	250	1	2	2	250z9	933068	75858	8.13	
9	[CC][3,5-ClSal]	250	1	2	3	250z9	795954	70999	8.92	
9	[CC][3,5-ClSal]	250	2	2	1	250z9	1047210	7016	0.67	
9	[CC][3,5-ClSal]	250	2	2	2	250z9	958910	16206	1.69	
9	[CC][3,5-ClSal]	250	2	2	3	250z9	760821	16282	2.14	
9	[CC][3,5-ClSal]	250	2	3	1	250z9	1019262	22424	2.20	
9	[CC][3,5-ClSal]	250	2	3	2	250z9	936964	49284	5.26	
9	[CC][3,5-ClSal]	250	2	3	3	250z9	771044	57983	7.52	
9	[CC][3,5-ClSal]	250	2	3	1	250z9	1013901	79388	7.83	
9	[CC][3,5-ClSal]	250	2	3	2	250z9	959429	78002	8.13	
9	[CC][3,5-ClSal]	250	2	3	3	250z9	794575	70876	8.92	3.17
10	3,5-dichlorosalicylic acid	250	1	1	1	250z10	1002206	37382	3.73	
10	3,5-dichlorosalicylic acid	250	1	1	2	250z10	932865	35822	3.84	
10	3,5-dichlorosalicylic acid	250	1	1	3	250z10	901344	44617	4.95	
10	3,5-dichlorosalicylic acid	250	1	1	1	250z10	1145840	64167	5.60	
10	3,5-dichlorosalicylic acid	250	1	1	2	250z10	1060462	80913	7.63	
10	3,5-dichlorosalicylic acid	250	1	1	3	250z10	721411	70843	9.82	
10	3,5-dichlorosalicylic acid	250	1	2	1	250z10	105998	10610	10.01	
10	3,5-dichlorosalicylic acid	250	1	2	2	250z10	897478	92440	10.30	

10	3,5-dichlorosalicylic acid	250	1	2	3	250z10	796866	93313	11.71	
10	3,5-dichlorosalicylic acid	250	2	2	1	250z10	1087502	40564	3.73	
10	3,5-dichlorosalicylic acid	250	2	2	2	250z10	915238	35145	3.84	
10	3,5-dichlorosalicylic acid	250	2	2	3	250z10	786874	38950	4.95	
10	3,5-dichlorosalicylic acid	250	2	3	1	250z10	1052702	58951	5.60	
10	3,5-dichlorosalicylic acid	250	2	3	2	250z10	934343	71290	7.63	
10	3,5-dichlorosalicylic acid	250	2	3	3	250z10	853465	83810	9.82	
10	3,5-dichlorosalicylic acid	250	2	3	1	250z10	1042991	104403	10.01	
10	3,5-dichlorosalicylic acid	250	2	3	2	250z10	946941	97535	10.30	
10	3,5-dichlorosalicylic acid	250	2	3	3	250z10	797688	93409	11.71	2.97
11	[CC][Isonicl]	250	1	1	1	250z11	1026853	185244	18.04	
11	[CC][Isonicl]	250	1	1	2	250z11	985114	180768	18.35	
11	[CC][Isonicl]	250	1	1	3	250z11	795192	146474	18.42	
11	[CC][Isonicl]	250	1	1	1	250z11	1125856	214926	19.09	
11	[CC][Isonicl]	250	1	1	2	250z11	928060	201203	21.68	
11	[CC][Isonicl]	250	1	1	3	250z11	774182	185184	23.92	
11	[CC][Isonicl]	250	1	2	1	250z11	1050928	253799	24.15	
11	[CC][Isonicl]	250	1	2	2	250z11	926495	228474	24.66	
11	[CC][Isonicl]	250	1	2	3	250z11	862246	215648	25.01	
11	[CC][Isonicl]	250	2	2	1	250z11	1200000	216480	18.04	
11	[CC][Isonicl]	250	2	2	2	250z11	947718	173906	18.35	
11	[CC][Isonicl]	250	2	2	3	250z11	787427	145044	18.42	
11	[CC][Isonicl]	250	2	3	1	250z11	1098089	209625	19.09	
11	[CC][Isonicl]	250	2	3	2	250z11	888120	192544	21.68	
11	[CC][Isonicl]	250	2	3	3	250z11	790888	189180	23.92	
11	[CC][Isonicl]	250	2	3	1	250z11	1076173	259896	24.15	
11	[CC][Isonicl]	250	2	3	2	250z11	963704	237649	24.66	
11	[CC][Isonicl]	250	2	3	3	250z11	762058	190591	25.01	2.92
12	isonicotinic acid	250	1	1	1	250z12	1024322	218898	21.37	
12	isonicotinic acid	250	1	1	2	250z12	916716	206261	22.50	

12	isonicotinic acid	250	1	1	3	250z12	799789	183712	22.97	
12	isonicotinic acid	250	1	1	1	250z12	1044617	243187	23.28	
12	isonicotinic acid	250	1	1	2	250z12	944465	241216	25.54	
12	isonicotinic acid	250	1	1	3	250z12	806880	225926	28.00	
12	isonicotinic acid	250	1	2	1	250z12	1029216	288901	28.07	
12	isonicotinic acid	250	1	2	2	250z12	943712	273205	28.95	
12	isonicotinic acid	250	1	2	3	250z12	816815	240879	29.49	
12	isonicotinic acid	250	2	2	1	250z12	1044890	223293	21.37	
12	isonicotinic acid	250	2	2	2	250z12	919627	206916	22.50	
12	isonicotinic acid	250	2	2	3	250z12	895778	205760	22.97	
12	isonicotinic acid	250	2	3	1	250z12	1067240	248453	23.28	
12	isonicotinic acid	250	2	3	2	250z12	917228	234260	25.54	
12	isonicotinic acid	250	2	3	3	250z12	838237	234706	28.00	
12	isonicotinic acid	250	2	3	1	250z12	1037055	291101	28.07	
12	isonicotinic acid	250	2	3	2	250z12	899628	260442	28.95	
12	isonicotinic acid	250	2	3	3	250z12	828827	244421	29.49	3.03
13	[CC][Sal]	250	1	1	1	250z13	1077576	89008	8.26	
13	[CC][Sal]	250	1	1	2	250z13	914197	79627	8.71	
13	[CC][Sal]	250	1	1	3	250z13	787251	76442	9.71	
13	[CC][Sal]	250	1	1	1	250z13	1090873	105924	9.71	
13	[CC][Sal]	250	1	1	2	250z13	915318	117435	12.83	
13	[CC][Sal]	250	1	1	3	250z13	787080	118770	15.09	
13	[CC][Sal]	250	1	2	1	250z13	970241	149417	15.40	
13	[CC][Sal]	250	1	2	2	250z13	934170	139285	14.91	
13	[CC][Sal]	250	1	2	3	250z13	789053	132955	16.85	
13	[CC][Sal]	250	2	2	1	250z13	1025744	84726	8.26	
13	[CC][Sal]	250	2	2	2	250z13	984058	85711	8.71	
13	[CC][Sal]	250	2	2	3	250z13	831966	80784	9.71	
13	[CC][Sal]	250	2	3	1	250z13	1027507	99771	9.71	
13	[CC][Sal]	250	2	3	2	250z13	888767	114029	12.83	

13	[CC][Sal]	250	2	3	3	250z13	797924	120407	15.09		
13	[CC][Sal]	250	2	3	1	250z13	1033554	159167	15.40		
13	[CC][Sal]	250	2	3	2	250z13	933557	139193	14.91		
13	[CC][Sal]	250	2	3	3	250z13	794208	133824	16.85	3.21	
**14**	**salicylic acid**	**250**	**1**	**1**	**1**	**250z14**	**623997**	**123895**	**19.86**		**Fig. S5**
14	salicylic acid	250	1	1	2	250z14	952058	214118	22.49		
14	salicylic acid	250	1	1	3	250z14	925644	209566	22.64		
14	salicylic acid	250	1	1	1	250z14	1000875	226998	22.68		
14	salicylic acid	250	1	1	2	250z14	816581	204880	25.09		
14	salicylic acid	250	1	1	3	250z14	744256	204670	27.50		
14	salicylic acid	250	1	2	1	250z14	1055781	293402	27.79		
14	salicylic acid	250	1	2	2	250z14	984131	260795	26.50		
14	salicylic acid	250	1	2	3	250z14	782483	220347	28.16		
14	salicylic acid	250	2	2	1	250z14	1068383	227352	21.28		
14	salicylic acid	250	2	2	2	250z14	926996	208481	22.49		
14	salicylic acid	250	2	2	3	250z14	863772	195558	22.64		
14	salicylic acid	250	2	3	1	250z14	1010699	229227	22.68		
14	salicylic acid	250	2	3	2	250z14	837985	210250	25.09		
14	salicylic acid	250	2	3	3	250z14	713630	196248	27.50		
14	salicylic acid	250	2	3	1	250z14	1029936	286219	27.79		
14	salicylic acid	250	2	3	2	250z14	994496	277663	27.92		
14	salicylic acid	250	2	3	3	250z14	832665	234478	28.16	2.80	
15	[CC][Nic]	250	1	1	1	250z15	1050617	90563	8.62		
15	[CC][Nic]	250	1	1	2	250z15	966631	84870	8.78		
15	[CC][Nic]	250	1	1	3	250z15	835294	73924	8.85		
15	[CC][Nic]	250	1	1	1	250z15	1118329	101097	9.04		
15	[CC][Nic]	250	1	1	2	250z15	888873	98487	11.08		
15	[CC][Nic]	250	1	1	3	250z15	819969	111762	13.63		
15	[CC][Nic]	250	1	2	1	250z15	1029642	143326	13.92		
15	[CC][Nic]	250	1	2	2	250z15	955207	140224	14.68		

15	[CC][Nic]	250	1	2	3	250z15	800902	120456	15.04	
15	[CC][Nic]	250	2	2	1	250z15	1083764	93420	8.62	
15	[CC][Nic]	250	2	2	2	250z15	951082	83505	8.78	
15	[CC][Nic]	250	2	2	3	250z15	781320	69147	8.85	
15	[CC][Nic]	250	2	3	1	250z15	1068128	96559	9.04	
15	[CC][Nic]	250	2	3	2	250z15	912874	101146	11.08	
15	[CC][Nic]	250	2	3	3	250z15	785721	107094	13.63	
15	[CC][Nic]	250	2	3	1	250z15	1027309	143001	13.92	
15	[CC][Nic]	250	2	3	2	250z15	934373	137166	14.68	
15	[CC][Nic]	250	2	3	3	250z15	799994	120319	15.04	2.70
16	nicotinic acid	250	1	1	1	250z16	1066391	184379	17.29	
16	nicotinic acid	250	1	1	2	250z16	928160	179599	19.35	
16	nicotinic acid	250	1	1	3	250z16	788559	156687	19.87	
16	nicotinic acid	250	1	1	1	250z16	1103537	220376	19.97	
16	nicotinic acid	250	1	1	2	250z16	952764	209513	21.99	
16	nicotinic acid	250	1	1	3	250z16	818706	196899	24.05	
16	nicotinic acid	250	1	2	1	250z16	1113073	278491	25.02	
16	nicotinic acid	250	1	2	2	250z16	964692	245900	25.49	
16	nicotinic acid	250	1	2	3	250z16	794994	203280	25.57	
16	nicotinic acid	250	2	2	1	250z16	1076539	186134	17.29	
16	nicotinic acid	250	2	2	2	250z16	957436	185264	19.35	
16	nicotinic acid	250	2	2	3	250z16	840432	166994	19.87	
16	nicotinic acid	250	2	3	1	250z16	995087	198719	19.97	
16	nicotinic acid	250	2	3	2	250z16	948205	208510	21.99	
16	nicotinic acid	250	2	3	3	250z16	814255	195828	24.05	
16	nicotinic acid	250	2	3	1	250z16	1032369	258299	25.02	
16	nicotinic acid	250	2	3	2	250z16	929955	237046	25.49	
16	nicotinic acid	250	2	3	3	250z16	770003	196890	25.57	2.99
1	[CC][2,6-diClIsonic]	125	1	1	1	125z1	1098900	85934	7.82	
1	[CC][2,6-diClIsonic]	125	1	1	2	125z1	942302	94796	10.06	

1	[CC][2,6-diClIsonic]	125	1	1	3	125z1	844721	93595	11.08	
1	[CC][2,6-diClIsonic]	125	1	1	1	125z1	1027824	121592	11.83	
1	[CC][2,6-diClIsonic]	125	1	1	2	125z1	945726	144980	15.33	
1	[CC][2,6-diClIsonic]	125	1	1	3	125z1	829144	152231	18.36	
1	[CC][2,6-diClIsonic]	125	1	2	1	125z1	1047353	194075	18.53	
1	[CC][2,6-diClIsonic]	125	1	2	2	125z1	914315	169971	18.59	
1	[CC][2,6-diClIsonic]	125	1	2	3	125z1	799558	172545	21.58	
1	[CC][2,6-diClIsonic]	125	2	2	1	125z1	998192	78059	7.82	
1	[CC][2,6-diClIsonic]	125	2	2	2	125z1	911174	91664	10.06	
1	[CC][2,6-diClIsonic]	125	2	2	3	125z1	762143	84445	11.08	
1	[CC][2,6-diClIsonic]	125	2	3	1	125z1	1061580	125585	11.83	
1	[CC][2,6-diClIsonic]	125	2	3	2	125z1	969790	148669	15.33	
1	[CC][2,6-diClIsonic]	125	2	3	3	125z1	759171	139384	18.36	
1	[CC][2,6-diClIsonic]	125	2	3	1	125z1	1000102	185319	18.53	
1	[CC][2,6-diClIsonic]	125	2	3	2	125z1	919077	170856	18.59	
1	[CC][2,6-diClIsonic]	125	2	3	3	125z1	763978	164866	21.58	4.62
2	2,6-dichloroisonicotinic acid	125	1	1	1	125z2	974298	132115	13.56	
2	2,6-dichloroisonicotinic acid	125	1	1	2	125z2	907028	131428	14.49	
2	2,6-dichloroisonicotinic acid	125	1	1	3	125z2	776107	113777	14.66	
2	2,6-dichloroisonicotinic acid	125	1	1	1	125z2	995915	146798	14.74	
2	2,6-dichloroisonicotinic acid	125	1	1	2	125z2	948651	169334	17.85	
2	2,6-dichloroisonicotinic acid	125	1	1	3	125z2	760215	159873	21.03	
2	2,6-dichloroisonicotinic acid	125	1	2	1	125z2	1091864	231038	21.16	
2	2,6-dichloroisonicotinic acid	125	1	2	2	125z2	956150	209684	21.93	
2	2,6-dichloroisonicotinic acid	125	1	2	3	125z2	775863	185664	23.93	
2	2,6-dichloroisonicotinic acid	125	2	2	1	125z2	1111863	150769	13.56	
2	2,6-dichloroisonicotinic acid	125	2	2	2	125z2	943178	136666	14.49	
2	2,6-dichloroisonicotinic acid	125	2	2	3	125z2	818635	120012	14.66	
2	2,6-dichloroisonicotinic acid	125	2	3	1	125z2	1010034	148879	14.74	
2	2,6-dichloroisonicotinic acid	125	2	3	2	125z2	901519	160921	17.85	

2	2,6-dichloroisonicotinic acid	125	2	3	3	125z2	817910	172006	21.03		
2	2,6-dichloroisonicotinic acid	125	2	3	1	125z2	1024934	216876	21.16		
2	2,6-dichloroisonicotinic acid	125	2	3	2	125z2	917909	201297	21.93		
2	2,6-dichloroisonicotinic acid	125	2	3	3	125z2	795786	190432	23.93	3.81	
3	[CC][3-ClSal]	125	1	1	1	125z3	1024952	2357	0.23		
3	[CC][3-ClSal]	125	1	1	2	125z3	946954	3977	0.42		
**3**	**[CC][3-ClSal]**	**125**	**1**	**1**	**3**	**125z3**	**906040**	**7123**	**0.78**		**Fig. S6**
3	[CC][3-ClSal]	125	1	1	1	125z3	1114139	10807	0.97		
3	[CC][3-ClSal]	125	1	1	2	125z3	977384	20721	2.12		
3	[CC][3-ClSal]	125	1	1	3	125z3	798200	27378	3.43		
3	[CC][3-ClSal]	125	1	2	1	125z3	1084354	39145	3.61		
3	[CC][3-ClSal]	125	1	2	2	125z3	928498	43918	4.73		
3	[CC][3-ClSal]	125	1	2	3	125z3	791246	52064	6.58		
3	[CC][3-ClSal]	125	2	2	1	125z3	1095100	2519	0.23		
3	[CC][3-ClSal]	125	2	2	2	125z3	969802	4073	0.42		
3	[CC][3-ClSal]	125	2	2	3	125z3	835034	5177	0.62		
3	[CC][3-ClSal]	125	2	3	1	125z3	1084775	10522	0.97		
3	[CC][3-ClSal]	125	2	3	2	125z3	976159	20695	2.12		
3	[CC][3-ClSal]	125	2	3	3	125z3	786087	26963	3.43		
3	[CC][3-ClSal]	125	2	3	1	125z3	1098695	39663	3.61		
3	[CC][3-ClSal]	125	2	3	2	125z3	917041	43376	4.73		
3	[CC][3-ClSal]	125	2	3	3	125z3	815624	53668	6.58	2.14	
4	3-chlorosalicylic acid	125	1	1	1	125z4	1237965	4580	0.37		
4	3-chlorosalicylic acid	125	1	1	2	125z4	1013890	36399	3.59		
4	3-chlorosalicylic acid	125	1	1	3	125z4	978562	35620	3.64		
4	3-chlorosalicylic acid	125	1	1	1	125z4	1214958	46897	3.86		
4	3-chlorosalicylic acid	125	1	1	2	125z4	1002359	51020	5.09		
4	3-chlorosalicylic acid	125	1	1	3	125z4	952209	37517	3.94		
4	3-chlorosalicylic acid	125	1	2	1	125z4	1227199	77927	6.35		
4	3-chlorosalicylic acid	125	1	2	2	125z4	1036313	72335	6.98		

4	3-chlorosalicylic acid	125	1	2	3	125z4	1000978	84983	8.49		
**4**	**3-chlorosalicylic acid**	**125**	**2**	**2**	**1**	**125z4**	**1124888**	**29451**	**2.62**		**Fig. S8**
4	3-chlorosalicylic acid	125	2	2	2	125z4	966255	34689	3.59		
4	3-chlorosalicylic acid	125	2	2	3	125z4	792443	28845	3.64		
4	3-chlorosalicylic acid	125	2	3	1	125z4	1061446	40972	3.86		
4	3-chlorosalicylic acid	125	2	3	2	125z4	918975	46776	5.09		
4	3-chlorosalicylic acid	125	2	3	3	125z4	767370	47117	6.14		
4	3-chlorosalicylic acid	125	2	3	1	125z4	1083675	68813	6.35		
4	3-chlorosalicylic acid	125	2	3	2	125z4	938231	65489	6.98		
4	3-chlorosalicylic acid	125	2	3	3	125z4	809169	68698	8.49	2.11	
5	[CC][4-ClSal]	125	1	1	1	125z5	1001979	84667	8.45		
5	[CC][4-ClSal]	125	1	1	2	125z5	971769	83475	8.59		
5	[CC][4-ClSal]	125	1	1	3	125z5	847227	75318	8.89		
5	[CC][4-ClSal]	125	1	1	1	125z5	963635	87016	9.03		
5	[CC][4-ClSal]	125	1	1	2	125z5	973971	117169	12.03		
5	[CC][4-ClSal]	125	1	1	3	125z5	816892	126373	15.47		
5	[CC][4-ClSal]	125	1	2	1	125z5	1193834	188506	15.79		
5	[CC][4-ClSal]	125	1	2	2	125z5	923439	149228	16.16		
5	[CC][4-ClSal]	125	1	2	3	125z5	801656	142935	17.83		
5	[CC][4-ClSal]	125	2	2	1	125z5	1057196	89333	8.45		
5	[CC][4-ClSal]	125	2	2	2	125z5	910689	78228	8.59		
5	[CC][4-ClSal]	125	2	2	3	125z5	819117	72820	8.89		
5	[CC][4-ClSal]	125	2	3	1	125z5	1111080	100331	9.03		
5	[CC][4-ClSal]	125	2	3	2	125z5	896126	107804	12.03		
5	[CC][4-ClSal]	125	2	3	3	125z5	828396	128153	15.47		
5	[CC][4-ClSal]	125	2	3	1	125z5	1045446	165076	15.79		
5	[CC][4-ClSal]	125	2	3	2	125z5	967393	156331	16.16		
5	[CC][4-ClSal]	125	2	3	3	125z5	779801	139039	17.83	3.73	
6	4-chlorosalicylic acid	125	1	1	1	125z6	1216902	150531	12.37		
6	4-chlorosalicylic acid	125	1	1	2	125z6	1009724	130860	12.96		

6	4-chlorosalicylic acid	125	1	1	3	125z6	841671	116656	13.86		
6	4-chlorosalicylic acid	125	1	1	1	125z6	1250532	174824	13.98		
6	4-chlorosalicylic acid	125	1	1	2	125z6	968048	166117	17.16		
6	4-chlorosalicylic acid	125	1	1	3	125z6	931371	190279	20.43		
6	4-chlorosalicylic acid	125	1	2	1	125z6	1227486	253353	20.64		
6	4-chlorosalicylic acid	125	1	2	2	125z6	954284	198014	20.75		
6	4-chlorosalicylic acid	125	1	2	3	125z6	559336	119586	21.38		
6	4-chlorosalicylic acid	125	2	2	1	125z6	981281	121384	12.37		
6	4-chlorosalicylic acid	125	2	2	2	125z6	960135	124433	12.96		
6	4-chlorosalicylic acid	125	2	2	3	125z6	873641	121087	13.86		
6	4-chlorosalicylic acid	125	2	3	1	125z6	1028316	143759	13.98		
6	4-chlorosalicylic acid	125	2	3	2	125z6	953979	163703	17.16		
6	4-chlorosalicylic acid	125	2	3	3	125z6	875083	178779	20.43		
6	4-chlorosalicylic acid	125	2	3	1	125z6	1064862	219788	20.64		
6	4-chlorosalicylic acid	125	2	3	2	125z6	926611	192272	20.75		
6	4-chlorosalicylic acid	125	2	3	3	125z6	849342	181589	21.38	3.68	
7	[CC][5-ClSal]	125	1	1	1	125z7	1116427	13955	1.25		
7	[CC][5-ClSal]	125	1	1	2	125z7	918109	11568	1.26		
**7**	**[CC][5-ClSal]**	**125**	**1**	**1**	**3**	**125z7**	**538827**	**12260**	**2.27**		**Fig. S7**
7	[CC][5-ClSal]	125	1	1	1	125z7	1080284	18473	1.71		
7	[CC][5-ClSal]	125	1	1	2	125z7	899143	31920	3.55		
7	[CC][5-ClSal]	125	1	1	3	125z7	860741	37700	4.38		
7	[CC][5-ClSal]	125	1	2	1	125z7	1132369	51410	4.54		
7	[CC][5-ClSal]	125	1	2	2	125z7	924328	46401	5.02		
7	[CC][5-ClSal]	125	1	2	3	125z7	860317	41381	4.81		
7	[CC][5-ClSal]	125	2	2	1	125z7	1079563	13495	1.25		
7	[CC][5-ClSal]	125	2	2	2	125z7	951140	11984	1.26		
7	[CC][5-ClSal]	125	2	2	3	125z7	880000	15752	1.79		
7	[CC][5-ClSal]	125	2	3	1	125z7	1022877	17491	1.71		
7	[CC][5-ClSal]	125	2	3	2	125z7	970779	34463	3.55		

7	[CC][5-ClSal]	125	2	3	3	125z7	754588	33051	4.38		
7	[CC][5-ClSal]	125	2	3	1	125z7	985954	44762	4.54		
7	[CC][5-ClSal]	125	2	3	2	125z7	964155	48401	5.02		
7	[CC][5-ClSal]	125	2	3	3	125z7	810138	38968	4.81	1.55	
8	5-chlorosalicylic acid	125	1	1	1	125z8	1394986	28039	2.01		
8	5-chlorosalicylic acid	125	1	1	2	125z8	1139224	24721	2.17		
**8**	**5-chlorosalicylic acid**	**125**	**1**	**1**	**3**	**125z8**	**686399**	**20466**	**2.98**		**Fig. S9**
8	5-chlorosalicylic acid	125	1	1	1	125z8	1265646	38855	3.07		
8	5-chlorosalicylic acid	125	1	1	2	125z8	1208192	50986	4.22		
8	5-chlorosalicylic acid	125	1	1	3	125z8	1028367	52035	5.06		
8	5-chlorosalicylic acid	125	1	2	1	125z8	1243817	69032	5.55		
8	5-chlorosalicylic acid	125	1	2	2	125z8	1213626	75123	6.19		
8	5-chlorosalicylic acid	125	1	2	3	125z8	995359	66988	6.73		
8	5-chlorosalicylic acid	125	2	2	1	125z8	1077518	21658	2.01		
8	5-chlorosalicylic acid	125	2	2	2	125z8	900927	19550	2.17		
8	5-chlorosalicylic acid	125	2	2	3	125z8	791634	24145	3.05		
8	5-chlorosalicylic acid	125	2	3	1	125z8	1111141	34112	3.07		
8	5-chlorosalicylic acid	125	2	3	2	125z8	926732	39108	4.22		
8	5-chlorosalicylic acid	125	2	3	3	125z8	821154	41550	5.06		
8	5-chlorosalicylic acid	125	2	3	1	125z8	1019472	56581	5.55		
8	5-chlorosalicylic acid	125	2	3	2	125z8	937518	58032	6.19		
8	5-chlorosalicylic acid	125	2	3	3	125z8	811900	54641	6.73	1.70	
9	[CC][3,5-ClSal]	125	1	1	1	125z9	1098144	61167	5.57		
9	[CC][3,5-ClSal]	125	1	1	2	125z9	935499	52762	5.64		
9	[CC][3,5-ClSal]	125	1	1	3	125z9	779277	49406	6.34		
9	[CC][3,5-ClSal]	125	1	1	1	125z9	1124257	79710	7.09		
9	[CC][3,5-ClSal]	125	1	1	2	125z9	951844	73197	7.69		
9	[CC][3,5-ClSal]	125	1	1	3	125z9	810073	69585	8.59		
9	[CC][3,5-ClSal]	125	1	2	1	125z9	1066352	102157	9.58		
9	[CC][3,5-ClSal]	125	1	2	2	125z9	940719	90403	9.61		

9	[CC][3,5-ClSal]	125	1	2	3	125z9	811283	81291	10.02	
9	[CC][3,5-ClSal]	125	2	2	1	125z9	1082938	60320	5.57	
9	[CC][3,5-ClSal]	125	2	2	2	125z9	935096	52739	5.64	
9	[CC][3,5-ClSal]	125	2	2	3	125z9	874407	55437	6.34	
9	[CC][3,5-ClSal]	125	2	3	1	125z9	1026674	72791	7.09	
9	[CC][3,5-ClSal]	125	2	3	2	125z9	935876	71969	7.69	
9	[CC][3,5-ClSal]	125	2	3	3	125z9	781401	67122	8.59	
9	[CC][3,5-ClSal]	125	2	3	1	125z9	972917	93205	9.58	
9	[CC][3,5-ClSal]	125	2	3	2	125z9	955540	91827	9.61	
9	[CC][3,5-ClSal]	125	2	3	3	125z9	820666	82231	10.02	1.69
10	3,5-dichlorosalicylic acid	125	1	1	1	125z10	1188830	121023	10.18	
10	3,5-dichlorosalicylic acid	125	1	1	2	125z10	1093509	119192	10.90	
10	3,5-dichlorosalicylic acid	125	1	1	3	125z10	940451	102697	10.92	
10	3,5-dichlorosalicylic acid	125	1	1	1	125z10	1213356	136503	11.25	
10	3,5-dichlorosalicylic acid	125	1	1	2	125z10	1071738	164405	15.34	
10	3,5-dichlorosalicylic acid	125	1	1	3	125z10	942998	175869	18.65	
10	3,5-dichlorosalicylic acid	125	1	2	1	125z10	1455611	273946	18.82	
10	3,5-dichlorosalicylic acid	125	1	2	2	125z10	1212080	231022	19.06	
10	3,5-dichlorosalicylic acid	125	1	2	3	125z10	991062	199798	20.16	
10	3,5-dichlorosalicylic acid	125	2	2	1	125z10	1056559	107558	10.18	
10	3,5-dichlorosalicylic acid	125	2	2	2	125z10	916267	99873	10.90	
10	3,5-dichlorosalicylic acid	125	2	2	3	125z10	772875	84398	10.92	
10	3,5-dichlorosalicylic acid	125	2	3	1	125z10	1085400	122108	11.25	
10	3,5-dichlorosalicylic acid	125	2	3	2	125z10	996671	152889	15.34	
10	3,5-dichlorosalicylic acid	125	2	3	3	125z10	831141	155008	18.65	
10	3,5-dichlorosalicylic acid	125	2	3	1	125z10	1166818	219595	18.82	
10	3,5-dichlorosalicylic acid	125	2	3	2	125z10	944983	180114	19.06	
10	3,5-dichlorosalicylic acid	125	2	3	3	125z10	835305	168397	20.16	4.09
11	[CC][Isonicl]	125	1	1	1	125z11	1085399	211327	19.47	
11	[CC][Isonicl]	125	1	1	2	125z11	920309	182405	19.82	

11	[CC][Isonicl]	125	1	1	3	125z11	864657	175352	20.28	
11	[CC][Isonicl]	125	1	1	1	125z11	1044055	216224	20.71	
11	[CC][Isonicl]	125	1	1	2	125z11	979133	237831	24.29	
11	[CC][Isonicl]	125	1	1	3	125z11	821866	231273	28.14	
11	[CC][Isonicl]	125	1	2	1	125z11	1015105	290929	28.66	
11	[CC][Isonicl]	125	1	2	2	125z11	883651	253519	28.69	
11	[CC][Isonicl]	125	1	2	3	125z11	798891	243981	30.54	
11	[CC][Isonicl]	125	2	2	1	125z11	1080791	210430	19.47	
11	[CC][Isonicl]	125	2	2	2	125z11	941262	186558	19.82	
11	[CC][Isonicl]	125	2	2	3	125z11	814713	165224	20.28	
11	[CC][Isonicl]	125	2	3	1	125z11	1115063	230930	20.71	
11	[CC][Isonicl]	125	2	3	2	125z11	979767	237985	24.29	
11	[CC][Isonicl]	125	2	3	3	125z11	775655	218269	28.14	
11	[CC][Isonicl]	125	2	3	1	125z11	1090198	312451	28.66	
11	[CC][Isonicl]	125	2	3	2	125z11	963588	276453	28.69	
11	[CC][Isonicl]	125	2	3	3	125z11	853825	260758	30.54	4.39
12	isonicotinic acid	125	1	1	1	125z12	1123983	301677	26.84	
12	isonicotinic acid	125	1	1	2	125z12	963423	259161	26.90	
12	isonicotinic acid	125	1	1	3	125z12	828184	222947	26.92	
12	isonicotinic acid	125	1	1	1	125z12	1022538	277312	27.12	
12	isonicotinic acid	125	1	1	2	125z12	951439	293138	30.81	
12	isonicotinic acid	125	1	1	3	125z12	765641	266367	34.79	
12	isonicotinic acid	125	1	2	1	125z12	1179804	411516	34.88	
12	isonicotinic acid	125	1	2	2	125z12	1027041	359362	34.99	
12	isonicotinic acid	125	1	2	3	125z12	823670	303440	36.84	
12	isonicotinic acid	125	2	2	1	125z12	1020882	274005	26.84	
12	isonicotinic acid	125	2	2	2	125z12	1017347	273666	26.90	
12	isonicotinic acid	125	2	2	3	125z12	718347	193379	26.92	
12	isonicotinic acid	125	2	3	1	125z12	1021387	277000	27.12	
12	isonicotinic acid	125	2	3	2	125z12	970868	299124	30.81	

12	isonicotinic acid	125	2	3	3	125z12	789437	274645	34.79	
12	isonicotinic acid	125	2	3	1	125z12	1073228	374342	34.88	
12	isonicotinic acid	125	2	3	2	125z12	972430	340253	34.99	
12	isonicotinic acid	125	2	3	3	125z12	773406	284923	36.84	4.13
13	[CC][Sal]	125	1	1	1	125z13	1062919	160501	15.10	
13	[CC][Sal]	125	1	1	2	125z13	967483	148122	15.31	
13	[CC][Sal]	125	1	1	3	125z13	852625	133862	15.70	
13	[CC][Sal]	125	1	1	1	125z13	1013444	159921	15.78	
13	[CC][Sal]	125	1	1	2	125z13	904884	182063	20.12	
13	[CC][Sal]	125	1	1	3	125z13	840543	199545	23.74	
13	[CC][Sal]	125	1	2	1	125z13	989604	237010	23.95	
13	[CC][Sal]	125	1	2	2	125z13	961869	233830	24.31	
13	[CC][Sal]	125	1	2	3	125z13	765692	198085	25.87	
13	[CC][Sal]	125	2	2	1	125z13	1060797	160180	15.10	
13	[CC][Sal]	125	2	2	2	125z13	974909	149259	15.31	
13	[CC][Sal]	125	2	2	3	125z13	853276	133964	15.70	
13	[CC][Sal]	125	2	3	1	125z13	1019429	160866	15.78	
13	[CC][Sal]	125	2	3	2	125z13	955875	192322	20.12	
13	[CC][Sal]	125	2	3	3	125z13	829161	196843	23.74	
13	[CC][Sal]	125	2	3	1	125z13	1112430	266427	23.95	
13	[CC][Sal]	125	2	3	2	125z13	950401	231042	24.31	
13	[CC][Sal]	125	2	3	3	125z13	732663	189540	25.87	4.41
14	salicylic acid	125	1	1	1	125z14	1094319	239109	21.85	
14	salicylic acid	125	1	1	2	125z14	912437	201831	22.12	
14	salicylic acid	125	1	1	3	125z14	838150	185483	22.13	
14	salicylic acid	125	1	1	1	125z14	1135290	251353	22.14	
14	salicylic acid	125	1	1	2	125z14	921001	247012	26.82	
14	salicylic acid	125	1	1	3	125z14	612842	186365	30.41	
14	salicylic acid	125	1	2	1	125z14	1240283	382751	30.86	
14	salicylic acid	125	1	2	2	125z14	954677	297000	31.11	

14	salicylic acid	125	1	2	3	125z14	841232	268942	31.97		
14	salicylic acid	125	2	2	1	125z14	1084181	236894	21.85		
**14**	**salicylic acid**	**125**	**2**	**2**	**2**	**125z14**	**542329**	**128386**	**23.67**		**Fig. S10**
14	salicylic acid	125	2	2	3	125z14	821077	181704	22.13		
14	salicylic acid	125	2	3	1	125z14	1069830	236860	22.14		
14	salicylic acid	125	2	3	2	125z14	900301	241461	26.82		
14	salicylic acid	125	2	3	3	125z14	763444	232163	30.41		
14	salicylic acid	125	2	3	1	125z14	1184805	365631	30.86		
14	salicylic acid	125	2	3	2	125z14	913443	284172	31.11		
14	salicylic acid	125	2	3	3	125z14	817584	261382	31.97	4.32	
15	[CC][Nic]	125	1	1	1	125z15	1205017	174848	14.51		
15	[CC][Nic]	125	1	1	2	125z15	934899	140422	15.02		
15	[CC][Nic]	125	1	1	3	125z15	722216	111149	15.39		
15	[CC][Nic]	125	1	1	1	125z15	1060140	165488	15.61		
15	[CC][Nic]	125	1	1	2	125z15	972467	190895	19.63		
15	[CC][Nic]	125	1	1	3	125z15	867711	201396	23.21		
15	[CC][Nic]	125	1	2	1	125z15	1002024	234774	23.43		
15	[CC][Nic]	125	1	2	2	125z15	900814	214214	23.78		
15	[CC][Nic]	125	1	2	3	125z15	775998	207657	26.76		
15	[CC][Nic]	125	2	2	1	125z15	1031319	149644	14.51		
15	[CC][Nic]	125	2	2	2	125z15	953840	143267	15.02		
15	[CC][Nic]	125	2	2	3	125z15	808687	124457	15.39		
15	[CC][Nic]	125	2	3	1	125z15	1014610	158381	15.61		
15	[CC][Nic]	125	2	3	2	125z15	920579	180710	19.63		
15	[CC][Nic]	125	2	3	3	125z15	812477	188576	23.21		
15	[CC][Nic]	125	2	3	1	125z15	1137555	266529	23.43		
15	[CC][Nic]	125	2	3	2	125z15	976030	232100	23.78		
15	[CC][Nic]	125	2	3	3	125z15	871144	233118	26.76	4.56	
16	Nicotinic acid	125	1	1	1	125z16	1048244	236274	22.54		
16	Nicotinic acid	125	1	1	2	125z16	985219	230738	23.42		

16	Nicotinic acid	125	1	1	3	125z16	740202	174096	23.52		
16	Nicotinic acid	125	1	1	1	125z16	1142692	272189	23.82		
16	Nicotinic acid	125	1	1	2	125z16	996296	273284	27.43		
16	Nicotinic acid	125	1	1	3	125z16	868845	271340	31.23		
16	Nicotinic acid	125	1	2	1	125z16	1136040	360693	31.75		
16	Nicotinic acid	125	1	2	2	125z16	992890	324973	32.73		
16	Nicotinic acid	125	1	2	3	125z16	841270	275516	32.75		
16	Nicotinic acid	125	2	2	1	125z16	1085472	244665	22.54		
16	Nicotinic acid	125	2	2	2	125z16	960752	225008	23.42		
16	Nicotinic acid	125	2	2	3	125z16	831145	195485	23.52		
16	Nicotinic acid	125	2	3	1	125z16	1120315	266859	23.82		
16	Nicotinic acid	125	2	3	2	125z16	984397	270020	27.43		
16	Nicotinic acid	125	2	3	3	125z16	782036	244230	31.23		
16	Nicotinic acid	125	2	3	1	125z16	1054677	334860	31.75		
16	Nicotinic acid	125	2	3	2	125z16	920113	301153	32.73		
16	Nicotinic acid	125	2	3	3	125z16	782645	256316	32.75	4.30	
0	UTC	250	1	1	1	UTC	1436174	450100	31.34		
0	UTC	250	1	1	2	UTC	1330371	418633	31.47		
0	UTC	250	1	1	3	UTC	1056718	332687	31.48		
0	UTC	250	1	1	1	UTC	919603	297421	32.34		
0	UTC	250	1	1	2	UTC	928109	320364	34.52		
0	UTC	250	1	1	3	UTC	655939	242172	36.92		
0	UTC	250	1	2	1	UTC	671413	250187	37.26		
0	UTC	250	1	2	2	UTC	1093067	409687	37.48		
0	**UTC**	**250**	**1**	**2**	**3**	**UTC**	**689900**	**265252**	**38.44**		**Fig. S11**
0	UTC	250	1	2	1	UTC	772990	241294	31.22		
0	UTC	250	2	2	2	UTC	1044808	330084	31.59		
0	UTC	250	2	2	3	UTC	918639	291111	31.69		
0	UTC	250	2	3	1	UTC	788915	256012	32.45		
0	UTC	250	2	3	2	UTC	994870	344226	34.60		

0	UTC	250	2	3	3	UTC	959611	353537	36.84	
0	UTC	250	2	3	1	UTC	796477	297175	37.31	
0	UTC	250	2	3	2	UTC	1025972	385353	37.56	
0	UTC	250	2	3	3	UTC	936331	357622	38.19	2.85

Examples of leaves treated with chlormequat-based ILs: total leaf area; area of necrotic spots and percentage of necrotic spot coverage

Figure S1. Induction of SAR triggered by spraying with [CC][3-ClSal] at a concentration of 250 mg/L. 1 -total leaf area; 2 – area of necrotic spots. Percentage of necrotic spot coverage = 0.27 %.

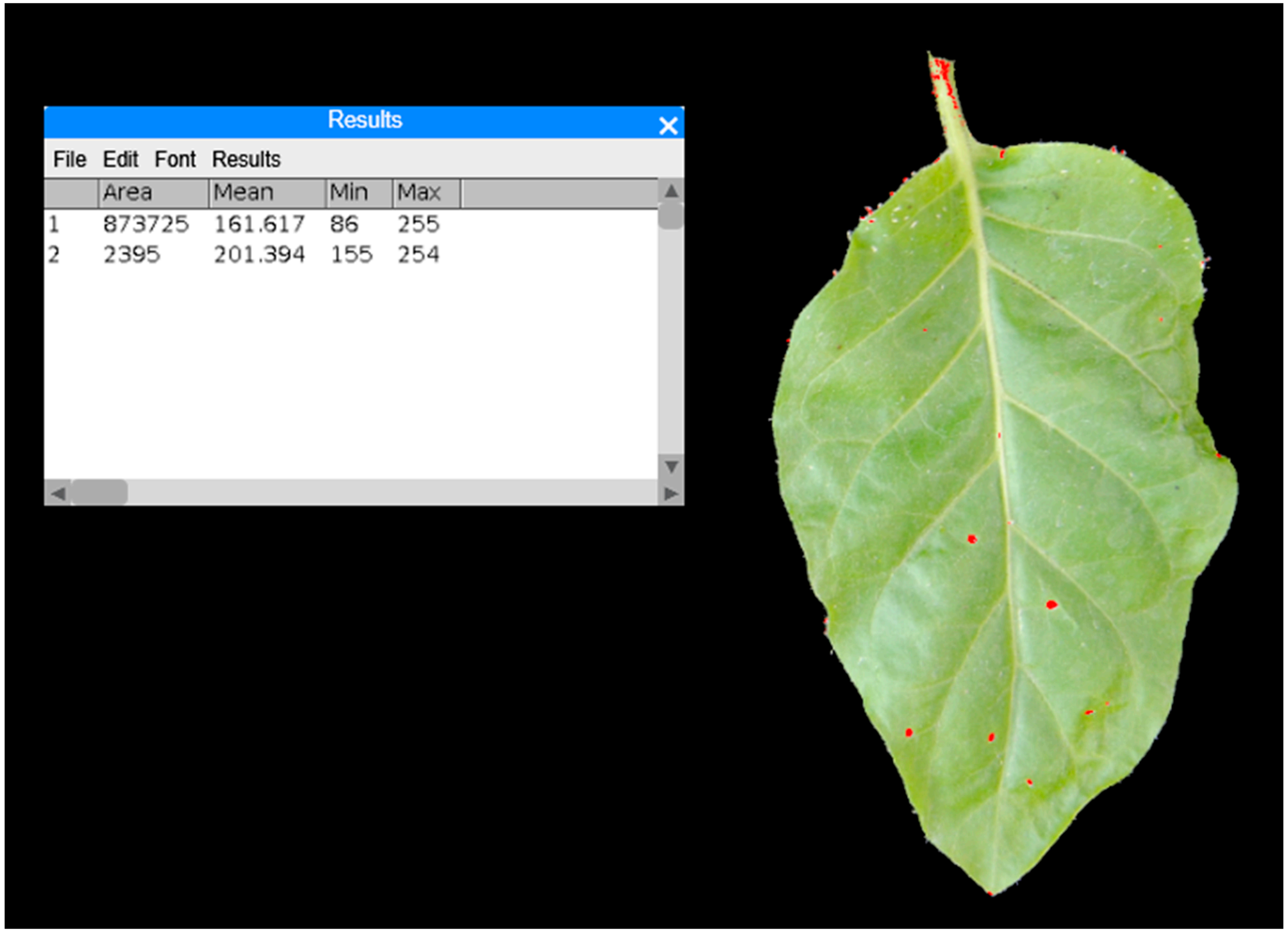

Figure S2. Induction of SAR triggered by spraying with [CC][5-ClSal] at a concentration of 250 mg/L. 1 -total leaf area; 2 – area of necrotic spots. Percentage of necrotic spot coverage = 1.15 %.

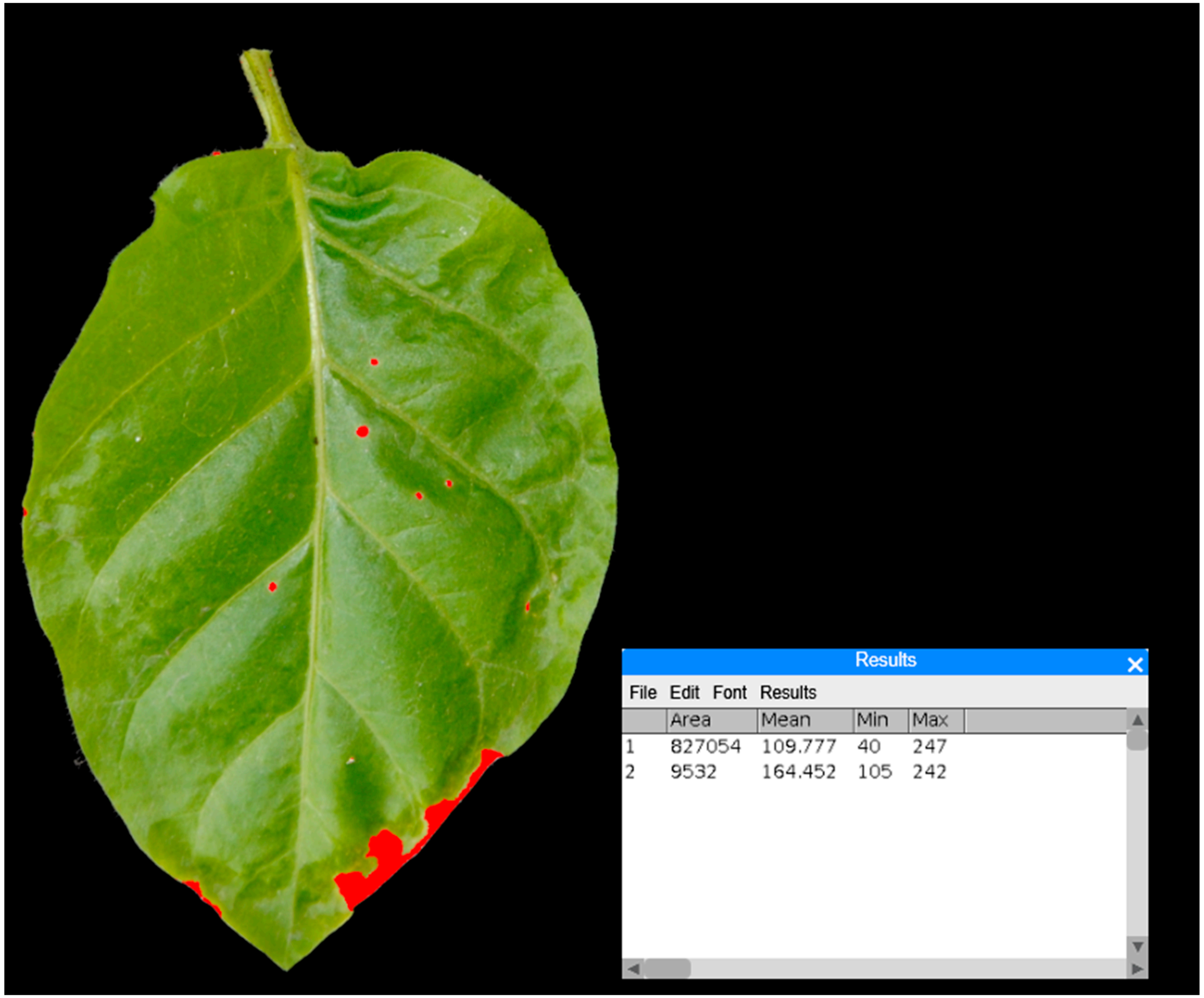

Figure S3. Induction of SAR triggered by spraying with 3-chlorosalicylic acid at a concentration of 250 mg/L. 1 -total leaf area; 2 – area of necrotic spots. Percentage of necrotic spot coverage = 2.30 %.

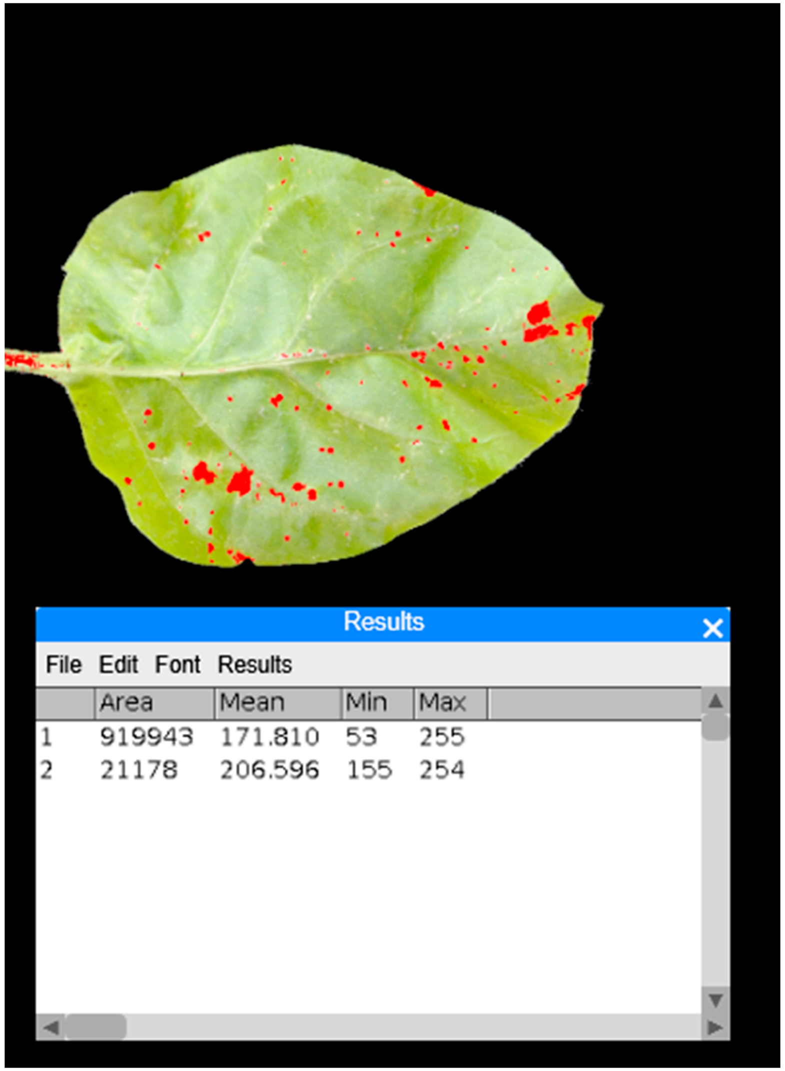

Figure S4. Induction of SAR triggered by spraying with 5-chlorosalicylic acid at a concentration of 250 mg/L. 1 -total leaf area; 2 – area of necrotic spots. Percentage of necrotic spot coverage = 1.28 %.

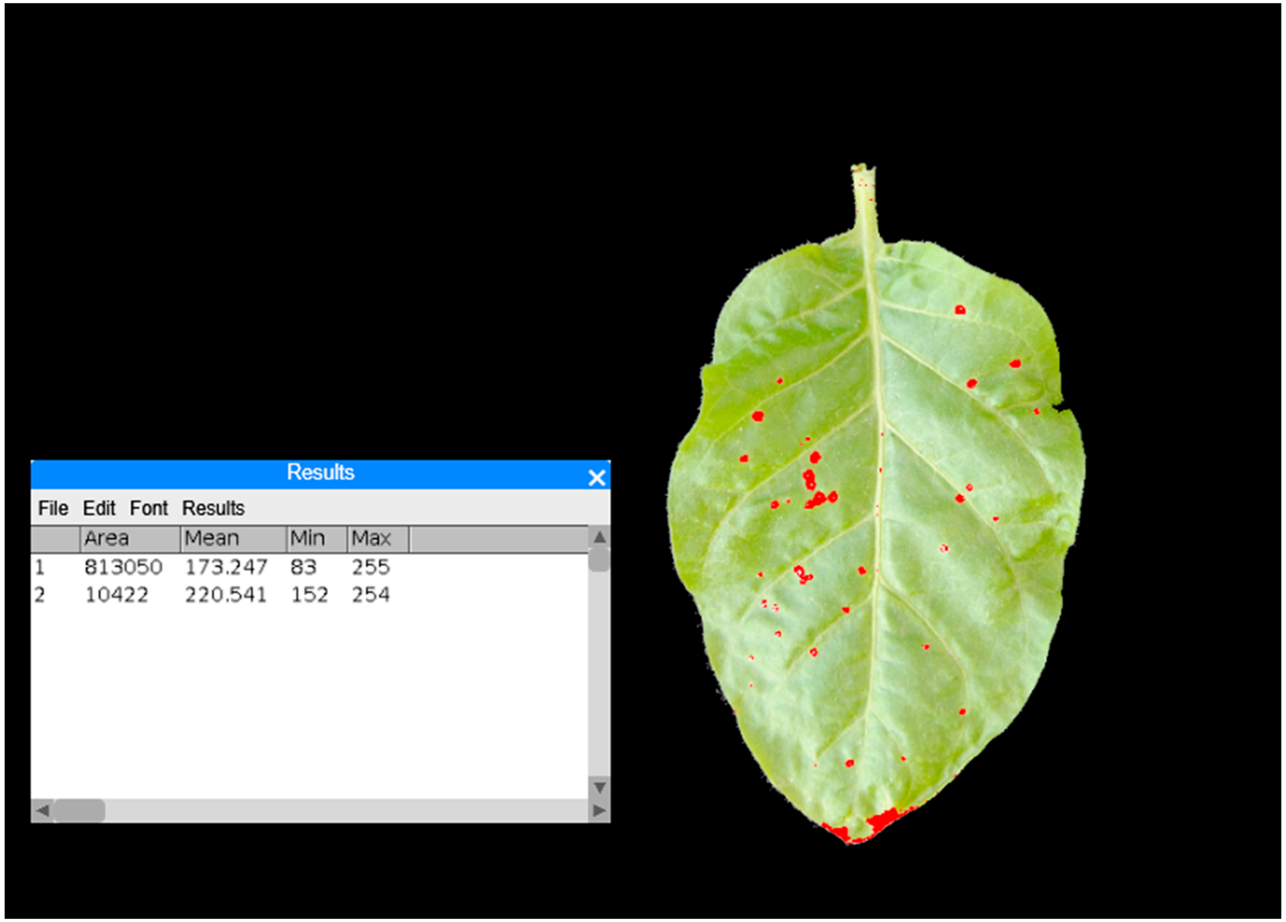

Figure S5. Induction of SAR triggered by spraying with salicylic acid at a concentration of 250 mg/L. 1 -total leaf area; 2 – area of necrotic spots. Percentage of necrotic spot coverage = 19.86 %.

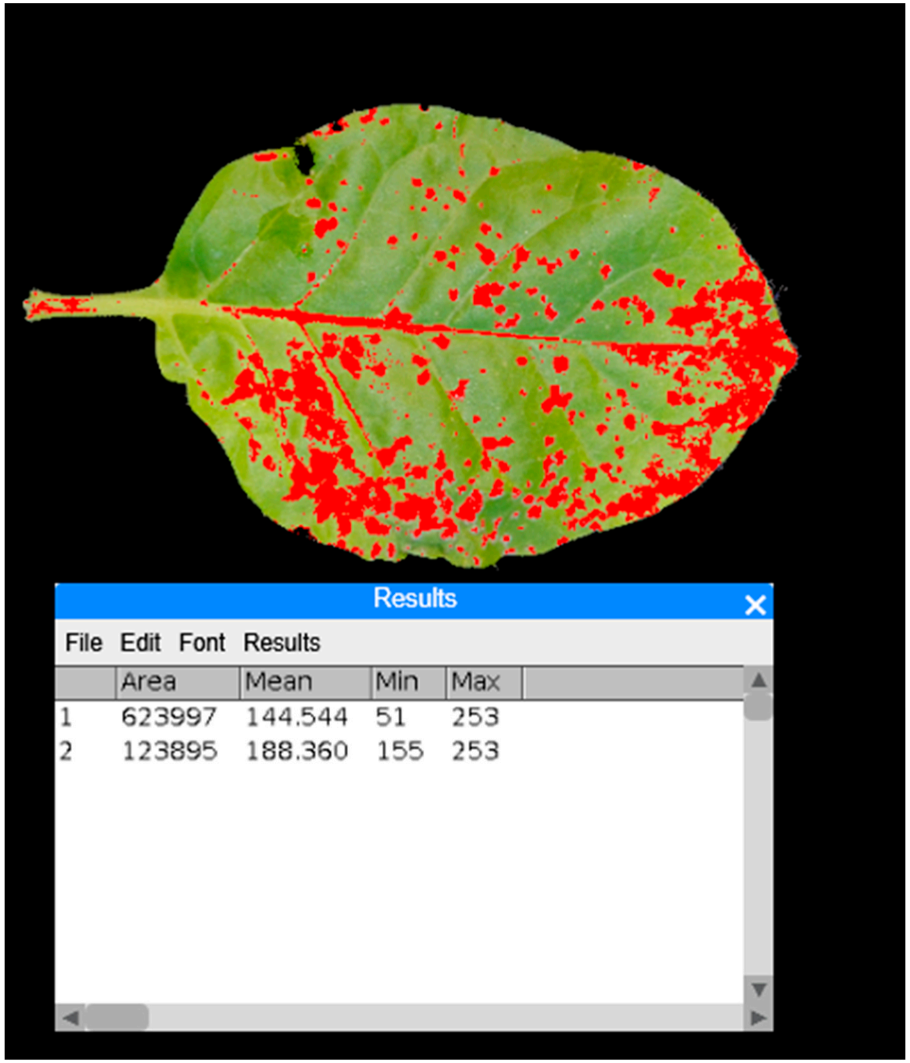

Figure S6. Induction of SAR triggered by spraying with [CC][3-ClSal] at a concentration of 125 mg/L. 1 -total leaf area; 2 – area of necrotic spots. Percentage of necrotic spot coverage = 0.79 %.

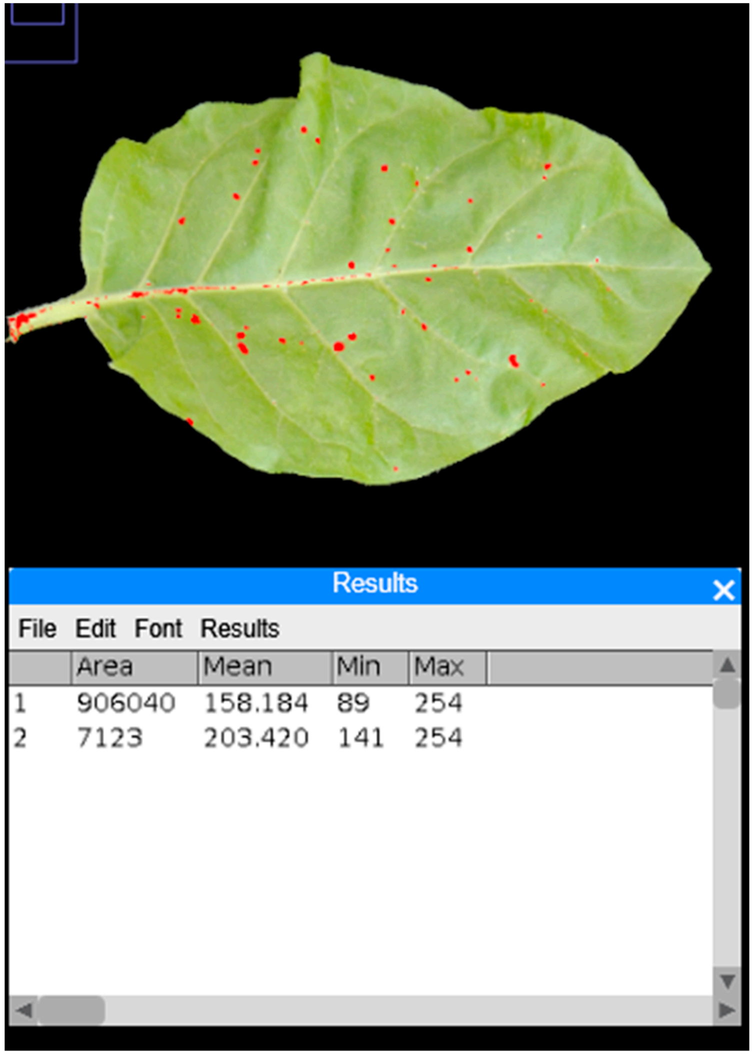

Figure S7. Induction of SAR triggered by spraying with [CC][5-ClSal] at a concentration of 125 mg/L. 1 -total leaf area; 2 – area of necrotic spots. Percentage of necrotic spot coverage = 2.28 %.

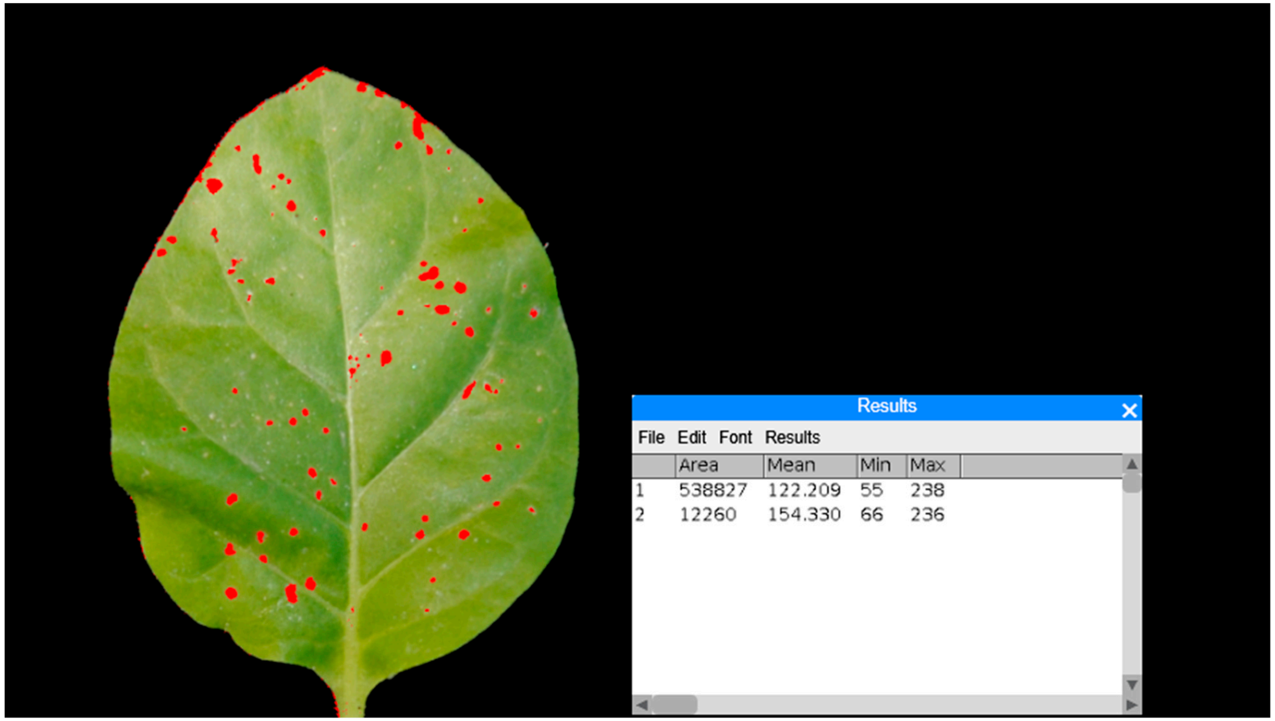

Figure S8. Induction of SAR triggered by spraying with 3-chlorosalicylic acid at a concentration of 125 mg/L. 1 -total leaf area; 2 – area of necrotic spots. Percentage of necrotic spot coverage = 2.62 %.

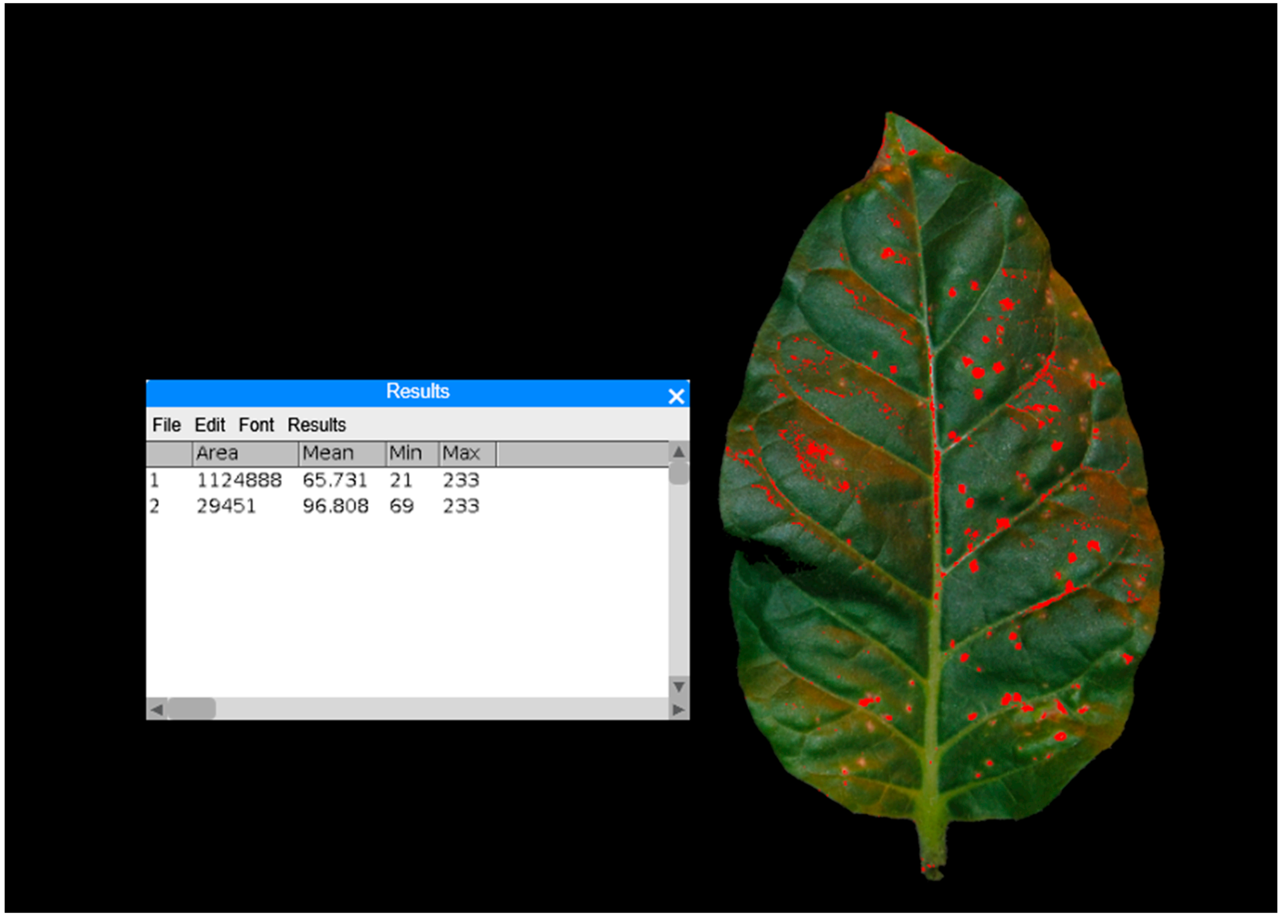

Figure S9. Induction of SAR triggered by spraying with 5-chlorosalicylic acid at a concentration of 125 mg/L. 1 -total leaf area; 2 – area of necrotic spots. Percentage of necrotic spot coverage = 2.98 %.

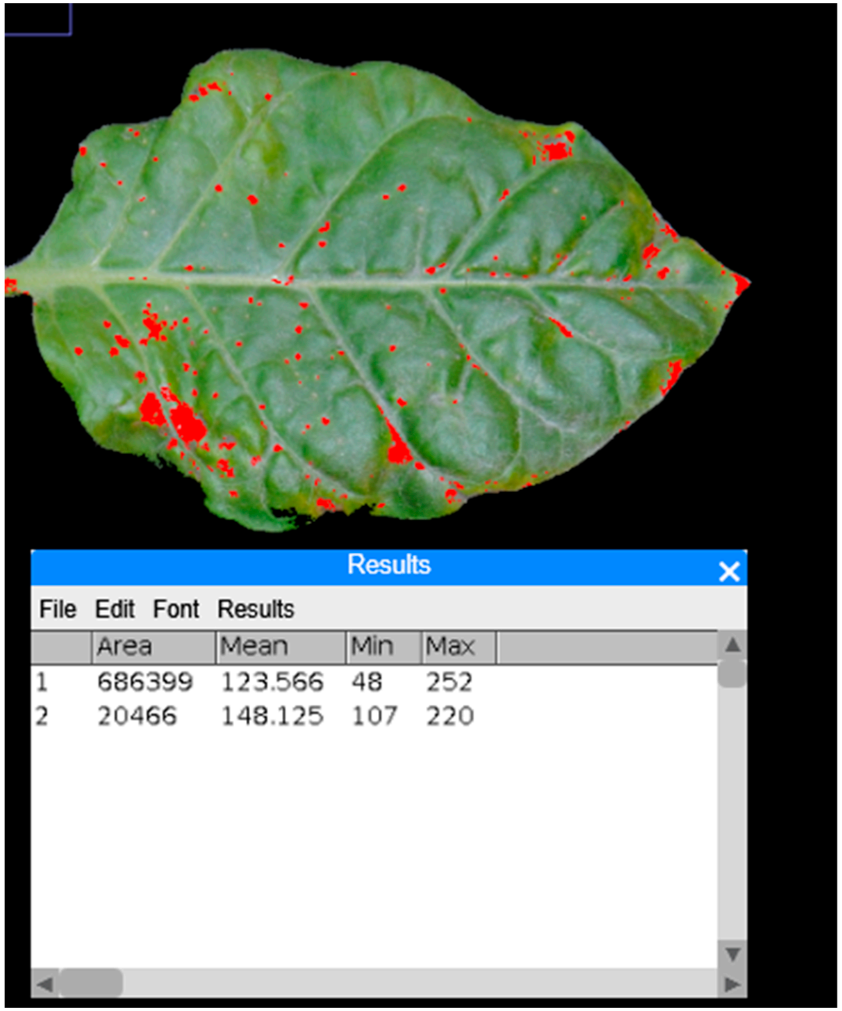

Figure S10. Induction of SAR triggered by spraying with salicylic acid at a concentration of 125 mg/L. 1 -total leaf area; 2 – area of necrotic spots. Percentage of necrotic spot coverage = 23.67 %.

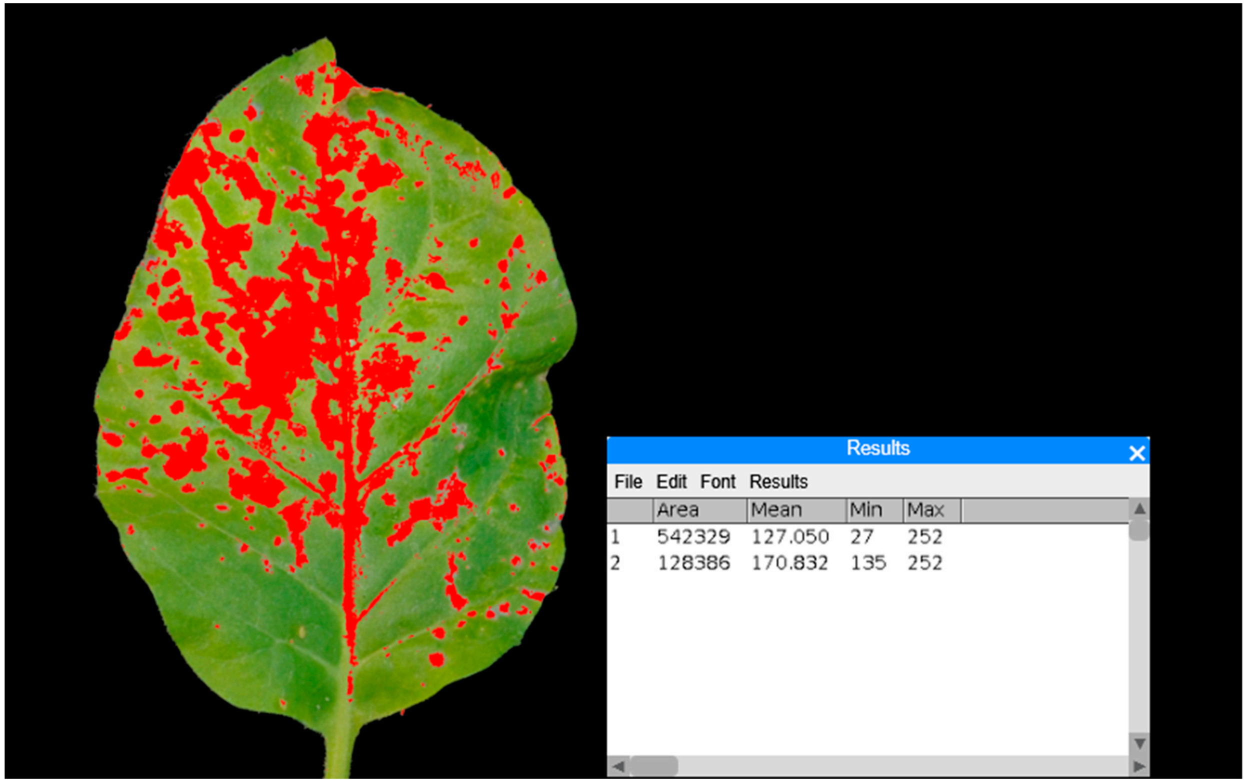

Figure S11. No induction of SAR effect – plants sprayed only with water (UTC). 1 -total leaf area; 2 – area of necrotic spots. Percentage of necrotic spot coverage = 38.44 %.

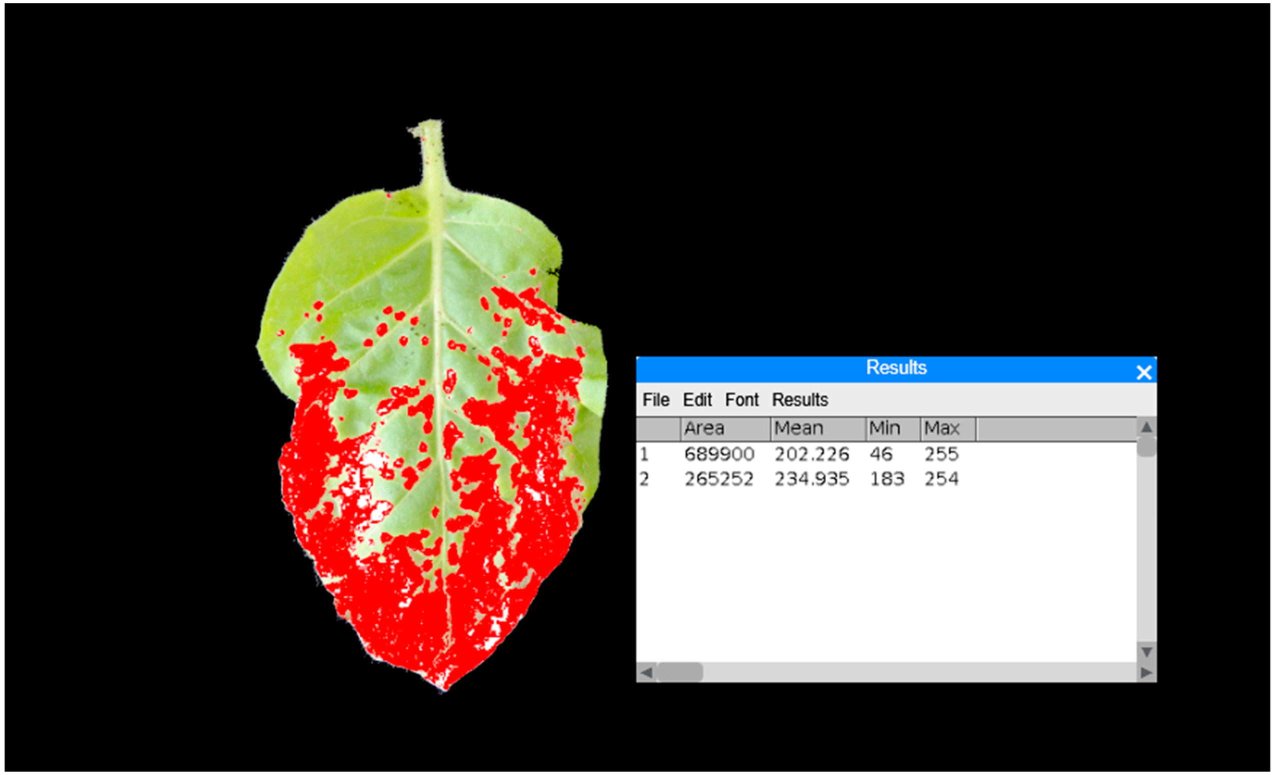

Supplement: Supplementary file 1 [file molecules-30-04203-s001.zip › molecules-3905408-supplementary.pdf]
